# mPEG-*b*-PCL-Based Nanoformulations of Aziridine-Functionalized 1,3,5-Triazine Derivative for Cancer Treatment

**DOI:** 10.3390/polym18192409

**Published:** 2026-10-02

**Authors:** Irina Bagaeva, Ekaterina Sinitsyna, Yuliia Zmitrichenko, Gregorii Tochilnikov, Elena Tupikina, Sergei Silonov, Mikhail Orlov, Viktor Korzhikov-Vlakh, Evgenia Korzhikova-Vlakh

**Affiliations:** 1 Institute of Chemistry, Saint-Petersburg State University, Universitetskiy Pr. 26, 198504 St. Petersburg, Russia; irina.bagaeva@spbu.ru (I.B.); e.tupikina@spbu.ru (E.T.); silonovsa25@yandex.ru (S.S.); st084341@student.spbu.ru (M.O.); v_korzhikov@mail.ru (V.K.-V.); 2 Branch of Petersburg Nuclear Physics Institute Named by B.P. Konstantinov of National Research Center “Kurchatov Institute”—Institute of Macromolecular Compounds, Bolshoi Pr. 31, 199004 St. Petersburg, Russia; kat_sinitsyna@mail.ru; 3 NMRC Institute of Oncology Named After N.N. Petrov, Leningradskaya Str. 68, Pesochny, 197758 St. Petersburg, Russia; zmitrichenko@gmail.com (Y.Z.); gr75@mail.ru (G.T.)

**Keywords:** poly(ethylene glycol)-*b*-poly(*ε*-caprolactone), nanoparticles, drug delivery systems, aziridine-functionalized triazine derivative, dioxadet, antitumor activity

## Abstract

Developing nanoformulations of antitumor drugs represents a critical priority in addressing key challenges of free drug administration such as low therapeutic efficacy, poor bioavailability, and severe side effects, including high systemic toxicity. In this study, mPEG-*b*-PCL block copolymers of different molecular weights were synthesized and applied to develop nanoformulations of the antitumor aziridine-functionalized 1,3,5-triazine derivative, dioxadet (DOD) via nanoprecipitation. The resulting DOD-loaded nanoparticles exhibited optimal characteristics for intravenous administration, and high encapsulation efficiency. The effect of mPEG-*b*-PCL molecular weight on drug encapsulation and release was investigated, and the results were elucidated through computational analysis of the molecular binding mechanism between DOD and PCL. Release profiles showed a sustained biphasic pattern. Determination of DOD concentration in blood of healthy rats indicated that the nanoformulation extended DOD’s circulation time. The empty nanoparticles were well-tolerated by both normal and cancer cells. The nanoformulation maintained inhibitory activity against A549 cells with an *IC*_50_, comparable to that of free DOD. The antitumor efficacy and reduced toxicity of the optimized nanoformulation were demonstrated in mice using two ascitic tumor models (Ehrlich ascites carcinoma and L1210 lymphoid leukemia) and one solid tumor model (Lewis lung epidermoid carcinoma) with the greatest therapeutic effect observed against the solid tumor.

## 1. Introduction

1,3,5-Triazine derivatives, also known as *s*-triazines, are symmetrical compounds and represent one of three isomers of a six-membered ring with three nitrogen atoms alternating with carbon atoms. These compounds have been extensively studied as potential antimicrobial [1], antiviral [2,3], anti-inflammatory [4] and anticancer [5] agents.

One of a highly promising 1,3,5-triazine derivative for cancer therapy is aziridine-functionalized derivative, namely, [5-[[4,6-bis(aziridin-1-yl)-1,3,5-triazin-2-yl]amino]-2,2-dimethyl-1,3-dioxan-5-yl]methanol, also known as dioxadet (DOD) [6,7]. Its amphiphilic nature allows for administration as both oil and aqueous formulations. In a comprehensive study, Mikolaichuk et al. evaluated DOD’s cytotoxic, hemolytic, and antioxidant properties [8]. They reported high hemocompatibility and low human serum albumin (HSA) affinity for the compound. DOD exhibited dose-dependent cytotoxicity against a panel of cancer cell lines (A549, PA-1, T98G, SK-HEP-1, and PANC-1), showing the greatest potency against the A549 line. The authors proposed that anticancer mechanism of DOD primarily involves DNA damage and the inhibition of reactive oxygen species (ROS) production.

Preclinical studies of DOD exhibit a favorable safety profile, with myelotoxicity being its primary dose-limiting factor and other side effects remaining minimal. This contrasts sharply with cisplatin and related platinum drugs, which provide severe hematotoxic, ototoxic, neurotoxic, and nephrotoxic effects even upon intraperitoneal administration [9,10,11,12]. In rat models of ovarian cancer, DOD treatment increased lifespan by 63.1%, outperforming cisplatin (48.1%). Clinical studies further demonstrated the high efficacy of DOD against stage III-IV ovarian tumors. Administered intravenously or intraperitoneally at a single dose of 15 mg every 72–96 h (up to a total dose of 90–120 mg), DOD was effective even in patients whose prior treatment with other alkylating cytostatics had failed [6,13].

Like many other cytostatic agents, 1,3,5-triazine derivatives lack specificity, which limits the dose that can be safely administered [14,15]. Nanoformulations address these drawbacks enabling enhanced permeability and retention in the tumor, reducing systemic side effects, and allowing for a controlled, prolonged release to improve therapeutic efficacy [16,17,18,19]. This approach is well-established, with several nano-based delivery systems already approved for clinical use [20,21]. In the field of developing nanoforms of DOD, there is a study on the encapsulation of DOD into nanoparticles based on a non-biodegradable block copolymer of poly(ethylene glycol) and poly(methacrylic acid) cross-linked with ethylenediamine or cysteamine [22]. The resulting nanoparticles were 130 nm in size and demonstrated the higher in vitro *IC*_50_ values against glioma cells (204–564 μg/mL) compared to the free drug (34–214 μg/mL), indicating a trade-off between high drug loading and therapeutic potency. Recently, Semenov et al. reported the development of DOD nanoforms representing conjugates of DOD with graphene oxide (GO) [23]. Comprehensive characterization confirmed successful conjugation and demonstrated that the GO-DOD nanoparticles (~150 nm) were stable, well-dispersed, and hemocompatible. The conjugate exhibited enhanced cytotoxicity against cancer cell lines (e.g., PANC-1) compared to free DOD, while showing reduced toxicity toward normal cells. However, the non-biodegradability of the carbon material and the limited control over release imposed by covalent binding remain major obstacles to clinical translation.

Among other delivery systems, polymer nanoparticles formed from biocompatible and biodegradable copolymers are highly promising. Currently, aliphatic polyesters such as poly(lactic acid) (PLA), poly(lactide-*co*-glycolide) (PLGA), and poly(ε-caprolactone) (PCL) are approved for biomedical applications because they degrade into non-toxic metabolites, namely hydroxycarboxylic acids. Their favorable properties make them a material of choice for a wide range of drug delivery platforms, including nano- and microparticles and implantable materials [24,25,26,27,28]. PEGylation is a crucial strategy to overcome the inherent limitations of aliphatic polyester-based materials such as high hydrophobicity and low stability in complex media [29,30]. The most common technique for preparation of PEGylated delivery systems is the formation of nanoparticles from block-copolymers of PEG and PLA, PLGA and PCL (PEG-*b*-PLA, PEG-*b*-PLGA, PEG-*b*-PCL) [31,32,33]. In contrast to PLA, PCL is more hydrophobic and semicrystalline, resulting in a longer degradation time [34,35]. Comparison of mPEG-*b*-PLA and mPEG-*b*-PCL nanoparticles have revealed higher colloidal stability of mPEG-*b*-PCL nanoparticles [36,37]. Numerous studies have demonstrated the efficacy of nano- and microparticles based on these copolymers for drug delivery and carriers for immunization [38,39,40,41,42,43]. For instance, Baghersad et al. reported the development of PCL-PAMAM C4 nanocarrier for co-delivery of silver nanoparticles and quercetin to breast cancer cells [44]. Wang et al. developed PEG-*b*-PCL polymersomes loaded with a curcumol derivative to enhance the drug’s bioavailability and its tumor growth-inhibitory effect [45]. However, there is a lack of studies which examined the influence of polymer molecular weight on drug loading, release, and efficacy.

**Figure 1:**
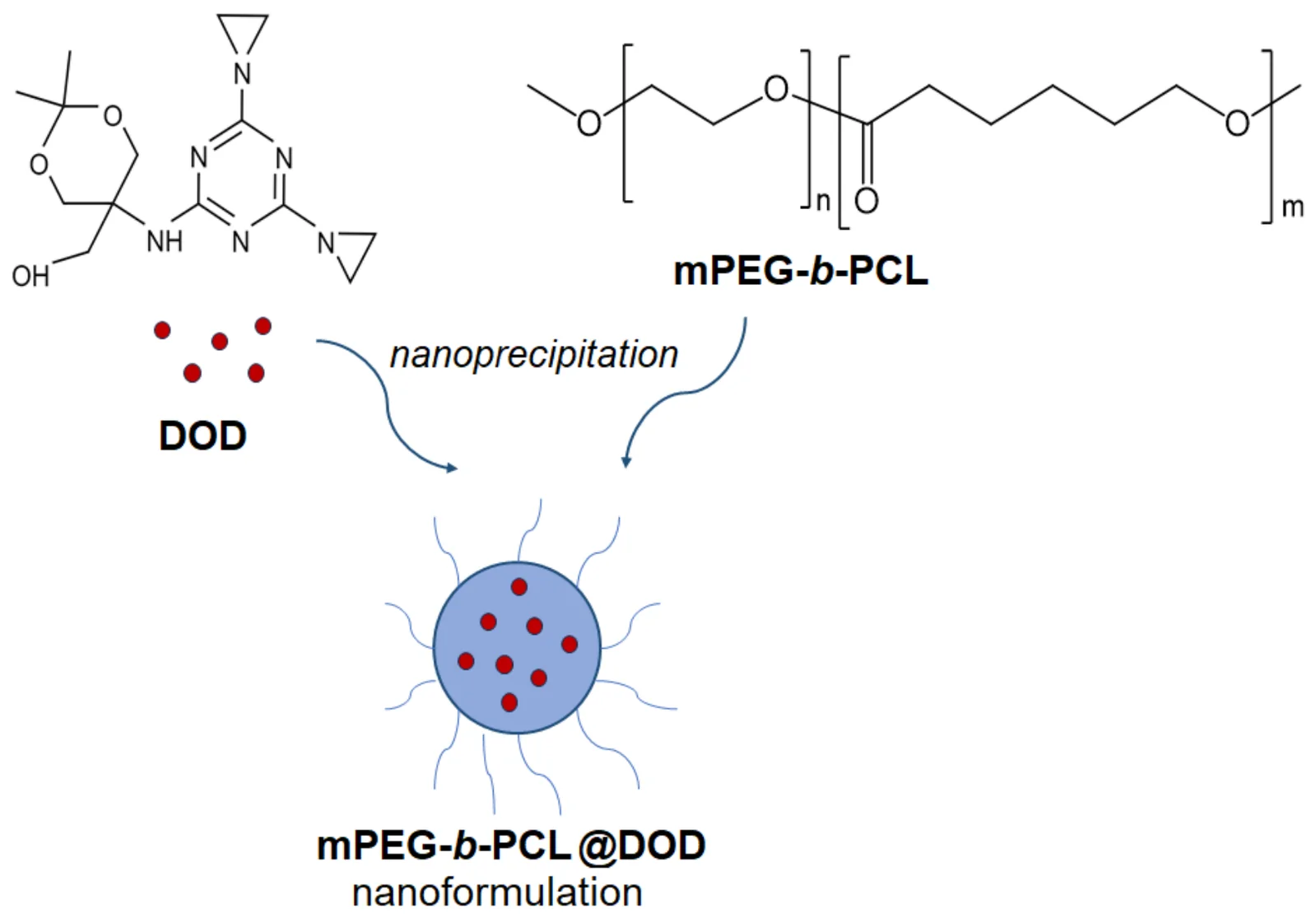
Chemical structures of the drug (DOD) and polymer (mPEG-*b*-PCL) used for the preparation of mPEG-*b*-PCL@DOD nanoformulation.

This study focuses on developing and optimizing a DOD nanoformulation using mPEG-*b*-PCL and release as well as performing a comprehensive biological evaluation. Figure 1 shows the chemical structures of the drug and polymer used to prepare nanoformulation. In contrast to other reported DOD systems, a biodegradable PCL core of mPEG-*b*-PCL and a non-covalent DOD loading ensure predictable in vivo clearance and release behavior. The optimization included the evaluation of PCL-block length on the drug loading and encapsulation efficacy as well as drug release rate. Furthermore, computational analysis clarified the molecular binding mechanism between DOD and PCL. The antitumor efficacy of the mPEG-*b*-PCL@DOD nanoformulation was evaluated and compared to the free drug in both in vitro and in vivo studies. The in vitro assessment was performed to determine the half maximal inhibitory concentration (*IC*_50_) and analyze the cell cycle in human lung carcinoma cells (A549 cell line). Subsequently, in vivo efficacy was investigated using murine models of L1210 lymphoid leukemia in DBA mice, Ehrlich ascites carcinoma in BALB/c mice and Lewis lung epidermoid carcinoma in C57Bl/6 mice.

## 2. Materials and Methods

### 2.1. Materials

Poly(ethylene glycol) monomethyl ether (mPEG, *M*_n_ = 5000) and stannous 2-ethylhexanoate (Sn(Oct)_2_, tin(II) octoate) were purchased from Sigma-Aldrich (Darmstadt, Germany). ε-Caprolactone (CL) was procured from J&K Scientific (Shanghai, China). Dioxadet (2,2-Dimethyl-5-((4,6-diaziridinyl-1,3,5-triazin-2-yl)amino)-1,3-dioxan-5-methanol, DOD, 99% purity) was obtained from Chemconsult (St. Petersburg, Russia). All organic solvents were supplied by Vecton (St. Petersburg, Russia) and purified according to standard laboratory protocols. Acetonitrile (ACN) was acquired from J.T. Baker (Phillipsburg, NJ, USA) and used as received. Dialysis membranes with molecular weight cut-offs (MWCO) of 2 kDa and 100 kDa were sourced from Orange Scientific (Braine-l’Alleud, Belgium), while a Spectra/POR^®^ Biotech CE membrane (MWCO 300 kDa) was purchased from Serva (Heidelberg, Germany). Hydrophobic PTFE syringe filters (13 mm diameter, 0.45 µm pore size) were obtained from Millex Merck-Millipore (Burlington, MA, USA).

### 2.2. Cells

Human lung carcinoma cells (A549) were purchased from the Cell bank of Institute of Cytology of Russian Academy of Sciences (St. Petersburg, Russia). Dulbecco’s Modified Eagle Medium (DMEM) (Capricorn Scientific, Ebsdorfergrund, Germany) was used as a basal medium for cell cultivation.

### 2.3. Animals

Female BALB/c, female DBA and female C57Bl/6 mice as well as female Wistar rats were obtained from the breeding colony of the “Andreevka” Branch of the Federal State Budgetary Institution “Scientific Center for Biomedical Technologies” of the Federal Medical-Biological Agency (Moscow Region, Russia). The animals were housed under standard conditions in accordance with the Guide for the Care and Use of Laboratory Animals [46], and the regulations for the design, equipment, and maintenance of experimental biological clinics (vivaria) of the Russian Federation (Decree No. 51, 29 August 2014, SP 2.2.1.3218-14 “Sanitary and Epidemiological Requirements for the Design, Equipment, and Maintenance of Experimental Biological Clinics (Vivaria)”).

Prior to the study, laboratory animals were acclimated for 14 days in cages. During the acclimatization and experimental periods, the animals were housed in cages with 10 mice per cage. The cages were equipped with steel grid lids featuring a feed recess and a steel feed divider. “Rodent Bedding” made from softwood shavings, produced by Laboratorkorm LLC (Moscow, Russia) was used as bedding. Granulated feed for animal maintenance: standard complete pelleted compound feed intended for laboratory rodents and rabbits, manufactured by Laboratorkorm LLC (Moscow, Russia), was provided ad libitum in the feed recess of the steel grid cage lid. The feed was free from pathogenic microflora that could affect the study results. Drinking water was provided ad libitum in standard drinkers with steel spout lids. The animals were housed under standard vivarium conditions at a temperature of 18–26 °C, relative humidity of 30–70%, and a regulated 12/12-h light/dark cycle.

Throughout the acclimatization, daily animal inspections were conducted, including an assessment of general behavior and overall condition.

### 2.4. Methods

#### 2.4.1. Polymer Synthesis and Characterization

Samples of mPEG-*b*-PCL were obtained via in bulk ring-opening polymerization of ε-caprolactone. mPEG with an *M_n_* of 5000 was used as the macroinitiator. Weighed portions of stannous octoate and the macroinitiator were placed in a 50 mL round-bottom flask, followed by the addition of 5 mL of the monomer. The following component ratios were used at this stage: [ε-caprolactone]:[Sn(Oct)_2_] = 1000, [ε-caprolactone]:[mPEG] = 50–400. The reaction was carried out under an argon atmosphere, with the temperature varying from 120 to 130 °C. The synthesis time ranged from 4 to 24 h. Upon completion of the reaction, the system was cooled, and the resulting copolymer was dissolved in chloroform. The polymer solution was concentrated and precipitated into a 10-fold excess of cold diethyl ether. The precipitate was isolated on a Schott filter (No. 40), then dissolved in DMF and dialyzed against a DMF:water (1:1) system for 4 h, followed by dialysis against pure water for 24 h. The solution was then evaporated to a dry residue using a rotary evaporator with the addition of ethanol. The residue of mPEG-*b*-PCL was dissolved in chloroform, precipitated into a 10-fold excess of diethyl ether, isolated on a Schott filter (No. 40), and dried in a vacuum for 36 h.

The polymer structure was confirmed by ^1^H NMR spectroscopy (CHCl_3_, 25 °C) using a Bruker Avance III WB 400 MHz spectrometer (Karlsruhe, Germany). The key signals of mPEG-*b*-PCL were found in the ^1^H NMR spectra (δ, ppm): PCL: 4.08 ppm (t, *J* = 6.7 Hz, -CH_2_-O-), 2.33 ppm (t, *J* = 7.5 Hz, -C(O)-CH_2_-), 1.67 ppm (m, -CH_2_-), and 1.41 ppm (m, -CH_2_-); PEG: 3.67 (br s, -OCH_2_CH_2_O-), 3.37 (s, -OCH_3_).

The number-average (*M_n_*) and weight-average (*M_w_*) molecular weights, as well as the dispersity (*Ð*) of the polymer samples, were analyzed by size-exclusion chromatography (SEC). SEC analyses were performed using a Shimadzu LC-20 Prominence system (Kyoto, Japan) equipped with a refractive index detector (RID-10A) and two Agilent PLgel MIXED-D columns (5 µm, 7.5 × 300 mm; Agilent Technologies, Santa Clara, CA, USA) at a 40 °C. THF was used as the mobile phase at a flow rate of 1.0 mL/min. Molecular-weight characteristics were calculated using a calibration plot built with se of polystyrene standards (Agilent Technologies, Santa Clara, CA, USA and Waters, Milford, MA, USA) ranging in molecular weight from 2000 to 450,000.

#### 2.4.2. Production and Characterization of Nanoformulations

Polymer nanoparticles based on the mPEG-*b*-PCL were formed using the nanoprecipitation method from two polymer samples with *M_w_* = 18,600 (*Đ* = 1.13), *M_w_* = 27,600 (*Đ* = 1.48), and *M_w_* = 42,500 (*Đ* = 1.55). The concentration of the copolymer in tetrahydrofuran ranged from 13 to 40 mg/mL. The concentration of DOD in the organic phase varied from 5 to 17 mg/mL. The solution containing the copolymer and DOD was filtered through PTFE Millex syringe filters with a pore size of 0.45 µm and a diameter of 13 mm. The volume of the organic phase was 1.5 mL. The polymer solution was injected into an aqueous phase with a volume of 6 mL using a syringe pump at a rate of 2 mL/min under stirring at 650 rpm. To remove the organic solvent, the system was left for 24 h at 23 °C.

For biological experiments, a DOD solution with concentration of 2 mg/mL was prepared. This solution was used to administer a dose of 4MTD (maximum tolerated dose) to animals. By the dilution method, suspensions containing 1.5 mg/mL (3MTD), 1.0 mg/mL (2MTD), and 0.5 mg/mL (MTD) were prepared. All these samples were obtained using sterile water for injection (Solopharm, St. Petersburg, Russia) and sterile labware.

The hydrodynamic diameter of the nanoparticles and their size distribution were analyzed by dynamic light scattering (DLS), while the zeta potential was determined by electrophoretic light scattering (ELS). Measurements were performed in distilled water using a Zetasizer Nano ZS Malvern Panalytical particle analyzer (Malvern, UK) at a scattering angle of 173° and a sample concentration of 0.1 mg/mL. Each sample was analyzed at room temperature (*n* = 3).

The morphology of the nanoparticles was analyzed using transmission electron microscopy (TEM). The sample was deposited onto copper grids (300 mesh) with a carbon and formvar coating and stained with a 2% uranyl acetate solution. An analysis was performed with the use of Jeol JEM-2100 TEM microscope (Kyoto, Japan). The average nanoparticle diameter in the dry state was measured by processing TEM images (*n* = 4) with ImageJ software (version 1.53t, National Institutes of Health, Bethesda, MD, USA). The number of particles used to calculate the average diameter was at least 50.

#### 2.4.3. Analysis of DOD Loading

After DOD encapsulation, a 6 mL aliquot of the mPEG-*b*-PCL@DOD dispersion was transferred into dialysis tubing with a 1000 kDa molecular weight cut-off (MWCO) membrane. To remove free drug, dialysis was performed for 1 h against deionized water, with the water being replaced every 15 min. After dialysis, the samples were frozen at −36 °C and then lyophilized for 48 h. The lyophilized powder was dissolved in methylene chloride, and the concentration of the encapsulated drug was determined by spectrophotometric analysis at 260 nm, using a pre-established calibration curve for DOD.

#### 2.4.4. Computational Details

Geometry optimization of DOD and DOD:PCL clusters were performed using Gaussian16 [47] at the B3LYP/6-311++G(d,p) level of theory, with empirical dispersion correction (GD3BJ) [48]. Initial conformational space exploration employed the GOAT search algorithm implemented in ORCA 6.0 software suite [49] at GFN2-xTB level of theory [50]. The five lowest-energy aggregated structures for the DOD + four-unit PCL model were fully optimized at the B3LYP-GD3BJ/6-311++G(d,p) level of theory.

Topological analysis of the electron density and energy densities at intermolecular critical points was completed for each optimized structure using MultiWFN 3.8 [51]. Electrostatic potential and IRI maps were calculated using MultiWFN as well.

The molecular structures and graphical representations were prepared using GaussView, version 6.1 (Gaussian Inc., Pittsburgh, PA, USA).

#### 2.4.5. DOD Release Study

The drug release profile of DOD from the nanoparticles was investigated using a nanoformulation with a drug loading of 374 µg/mg of polymer. A number of parallel samples was equal to 3. A 750 µL aliquot of the nanoparticle dispersion was loaded into a dialysis tube (50–800 µL volume, 1000 kDa MWCO) and immersed into 12 mL of release medium (0.01 M PBS, pH 7.4). The assembly was incubated in a thermoshaker at 37 °C and 120 rpm. The external release medium was replaced at predetermined intervals: 1, 2, 4, 6, 24, 48, 96, 144, and 216 h (within 9 days). The collected samples were analyzed spectrophotometrically at 260 nm. The concentration of released DOD was quantified using a pre-built calibration curve in PBS.

#### 2.4.6. Analysis of DOD Concentration in Rat Blood

Study was conducted on female Wistar rats, three months of age at the start of the research. Each experimental group included 3 animals. The MTD of DOD in rats is 2.5 mg/kg. In all cases, the drugs were administered intraperitoneally in a volume of 0.2 mL as a single dose. Blood was drawn from the rats into vacuum tubes containing a coagulation activator on day 1, 3, 5, 7 and 10. The blood was then centrifuged at a centrifugal force of 1500 g (3000 rpm) for 15 min. The rats were divided into three groups according to the DOD dose received—2MTD/NPs, 3MTD/NPs and 4MTD/NPs.

Before the chromatographic analysis, proteins were removed from the serum samples. For this purpose, 200 μL of an acetonitrile/methanol mixture (1/1, *v*/*v*) was added to 100 μL of blood serum, and the resulting mixture was centrifuged at 15,000 rpm for 20 min. It was then frozen for 2 days and centrifuged again at 15,000 rpm for 20 min. After that, the supernatant was collected for chromatographic analysis by HPLC-MS.

DOD analysis was performed using a LCMS-8030 Shimadzu system (upgraded to LCMS-8030Plus) with a tandem triple quadrupole mass-selective detector (Tokyo, Japan) and equipped with a Zorbax Eclipse XDB-C18 column (150 mm × 2.1 mm, 5 µm, 100 Å) (Torrance, CA, USA). The volume of the injected sample was 1 µL. Water for chromatography (LC-MS Grade, LiChrosolv^®^, Merck KGaA, Darmstadt, Germany) (eluent A) (eluent A) and acetonitrile (LC-MS grade, Panreac, Barcelona, Spain) (eluent B) were used as eluents. Both eluents were acidified with 0.1% formic acid (Formic acid LC-MS grade, Carlo Erba, Val de Reuil, France). The analysis was performed using a gradient mode at 40 °C. The flow rate of the mobile phase was 0.4 mL/min. The following elution program was used for analysis: 0–1 min, 5% B; 1–8 min, from 5 to 40% B; 8–8.50 min, from 40 to 100% B; 8.5–12 min, 100% B; and 12–16 min, 5% B. 

#### 2.4.7. mPEG-*b*-PCL Cytotoxicity and DOD Inhibition Activity Studies

Cytotoxicity of empty nanoparticles and inhibitory activity of free DOD and its nanoformulation were investigated using an MTT assay on HEK 293 and A549 cell lines. The cells were seeded in 96-well plates at 3000 cells per well in 100 µL of DMEM growth medium containing 10% FBS (*v*/*v*), 0.292 g/L glutamine, 1 g/L glucose, and 0.05 g/L gentamicin. After cell seeding overnight, 100 µL of growth medium containing mPEG-*b*-PCL nanoparticles, mPEG-*b*-PCL@DOD nanoformulation, and free DOD were added to each well. For the empty nanoparticles, the concentrations ranged from 2–500 µg/mL. For DOD and its nanoformulation, from 0.25 to 70 µg/mL regarding DOD. The incubation was performed for 72 h at 37 °C in a 5% CO_2_ atmosphere.

After this period, 10 µL of an MTT solution with a concentration of 10 mg/mL was added to the wells and left for 1.5 h. Subsequently, the growth medium was removed, the formazan crystals were dissolved in 150 µL of DMSO, and the absorption of the solutions was measured. This included untreated cells and cell-free wells as controls, measured at wavelengths of 555 nm and 670 nm. Measurements were performed using a SPECTROstar Nano microplate reader (BMG Labtech, Saitama, Germany). Data processing was carried out using Origin 2018 software.

#### 2.4.8. Cell Cycle Study

Cell cycle analysis was performed by flow cytometry using the A549 cell line. With this aim, cells were seeded in 6-well plates at 75,000 cells per well in 1 mL of DMEM growth medium containing 10% FBS (*v*/*v*), 0.292 g/L glutamine, 1 g/L glucose, and 0.05 g/L gentamicin. After incubation overnight, 1 mL of growth medium containing mPEG-*b*-PCL nanoparticles (2 µg/mL), mPEG-*b*-PCL@DOD nanoformulation (0.05-10 µg/mL), or free DOD (2 µg/mL) was added to each well. The cells were incubated for 72 h at 37 °C in a 5% CO_2_ atmosphere. After that, the cells were detached from the plates by trypsinization, centrifuged and the supernatant was removed. The cells were resuspended in ice-cold 70% ethanol and fixed for 30 min at −20 °C, then centrifuged at 500 g for 5 min. The supernatant was removed, the cells were washed with Dulbecco’s PBS, and resuspended in 50 µL of an RNAse A solution at a concentration of 100 µg/mL. The cells were incubated for 10 min at 37 °C and then stained with propidium iodide (PI) for 10 min at a concentration of 10 µg/mL. Cell fluorescence was measured using a NovoCyte Advantion flow cytometer (Agilent, Santa Clara, CA, USA). Data processing was performed using NovoExpress software (Agilent, Santa Clara, CA, USA).

#### 2.4.9. Tumor Grafting to Mice

Animals were randomly assigned to experimental groups, ensuring that each individual’s body weight did not deviate from the group mean by more than 10%.

BALB/c mice were grafted with Ehrlich ascitic adenocarcinoma, while DBA mice were grafted with L1210 lymphoid leukemia. The study was conducted using three-month-old BALB/c (female) and DBA (female) mice. The animal with an ascitic transplantable tumor, euthanized in a chamber via CO_2_ inhalation, was fixed on a dissection table. The abdominal skin was treated with alcohol and a 3–5% iodine solution. The skin was incised with sterile scissors. The abdominal wall, exposed after skin removal, was carefully punctured with a medium-diameter needle, and ascitic fluid was aspirated into a syringe. The cell count in the ascitic fluid was determined, and it was diluted with sterile 0.9% sodium chloride solution to the required concentration per unit volume. The cell concentration per unit volume used for inoculation ranged from 1 × 10^6^ to 5 × 10^6^ cells. A volume of 0.2 mL was administered intraperitoneally to the mice. All manipulations with tumor tissue were performed under cooling conditions using cryogenic agents.

Female C57Bl/6 mice were intramuscularly transplanted with Lewis lung epidermoid carcinoma by injection of a 10% tumor cell suspension. A mouse with a solid transplantable tumor was euthanized and subsequently prepared using the way described above. Tumor fragments without visible necrosis were isolated and transferred into sterile glass or plastic containers. The fragments were then washed with sterile 0.9% sodium chloride solution, minced, and sequentially passed through a syringe using needles of progressively smaller diameters. The resulting homogenate was diluted with isotonic sodium chloride solution to achieve a uniform 10% tumor cell suspension. This suspension was administered intramuscularly in a volume of 0.2 mL per mouse. All manipulations with tumor tissue were performed under cooled conditions using cooling agents.

#### 2.4.10. Evaluation of Antitumor Activity In Vivo

The day of tumor transplantation was designated as day 0. Randomization and group labeling of the mice were performed on the day of tumor transplantation. The animals were identified using biological dyes for animal marking. Each experimental group included 10 animals. The control group consisted of 10 animals for the Ehrlich ascitic adenocarcinoma and lymphoid leukemia L1210 models, and 6 animals for the Lewis lung epidermoid carcinoma model. For all tumor models, treatment started 48 h after tumor grafting. The MTD of DOD in mice is 5 mg/kg. In all cases, the drugs were administered intraperitoneally in a volume of 0.2 mL as a single dose. A description of the experimental groups is shown in Table 1.

The antitumor activity of the drug is assessed based on tumor growth delay and tumor regression (using parameters such as tumor growth inhibition percentage and tumor growth index), increase in animal lifespan, percentage of cured animals, and hazard ratio [3]. The increase in lifespan (%) was calculated using Equation (1):

(1) $$\mathrm{Increase}\;\mathrm{in}\;\mathrm{lifespan}\;=\frac{{\mathrm{MST}}_{e}-{\mathrm{MST}}_{c}}{{\mathrm{MST}}_{c}}\times 100\%$$

where *MST_c_* is the median survival time of the control group, *MST_e_* is the median survival time of the experimental group.

In the case of Lewis lung epidermoid carcinoma, the following parameters were also evaluated: the tumor growth inhibition (*TGI*), tumor volume (*V_t_*), tumor growth index (*TG*), and the area under the tumor growth kinetic curve (*S*).

The *TGI* values (%) were calculated using Equation (2):

(2) $$\mathrm{TGI}\;=\;\frac{V_{c}-V_{e}}{V_{c}}\times 100\%$$

where *V_c_* is the mean tumor volume in the control group of mice; *V_e_* is the mean tumor volume in the experimental group of mice treated with the drug.

Tumor volume (mm^3^) was calculated using the ellipsoid Formula (3):

(3) $$V=\;\frac{A\cdot B^{2}\cdot \pi }{6}\approx \frac{A\cdot B^{2}}{2}$$

where *A* is the largest tumor diameter; B is the diameter perpendicular to A.

*TG* (%) was calculated using the following Equation (4):

(4) $$\mathrm{TG}\;=\;\frac{S_{e}}{S_{c}}\times 100\%$$

where *S_e_* is the area under the tumor growth kinetic curve for the group of mice that received therapy; *S_c_* is the area under the tumor growth kinetic curve for the control group of mice.

The area under the tumor growth kinetic curve was measured using the trapezoidal method, based on the Formula (5):

(5) $$S=\sum_{i=1}^{n-1}\frac{V_{i}+V_{i+1}}{2}\times t_{i}=\frac{V_{1}+V_{2}}{2}\times t_{1}=\frac{V_{2}+V_{3}}{2}\times t_{2}+\ldots +\frac{V_{n-1}+V_{n}}{2}\times t_{n-1}$$

where *V_i_* is the tumor volume at the corresponding measurement point *i*; *n* is the total number of measurements; *t*_1_ is the time in days between the first and second measurements; *t*_2_ is the time in days between the second and third measurements; *t_n−_*_1_ is the time in days between the penultimate and last measurements.

#### 2.4.11. Statistical Analysis

GraphPad Prism software (version 8.0.1) was applied for the statistical analysis of MTT-assay data. A comparison between the two samples (control and treated) was performed via the two-way analysis of variances (ANOVA). Data were processed using SPSS Statistics 26.0 software (IBM, Armonk, NY, USA). The arithmetic mean and standard error of the mean (SEM) were calculated. Differences between groups were assessed using the Mann-Whitney U-test and Fisher’s exact test. Data visualization was performed using GraphPad Prism software. Overall animal survival data are graphically presented as Kaplan–Meier curves. A *p*-value < 0.05 was considered statistically significant. Hazard ratio (HR) and its 95% confidence interval (95% CI) were calculated using *Cox* regression analysis.

## 3. Results and Discussion

### 3.1. Polymer Synthesis

In this study, mPEG with number-average molecular weight of 5000 was selected for the synthesis of mPEG-*b*-PCL. According to the literature this PEG chain length is suitable for providing effective stabilization of nanoparticles [52,53]. In turn, PCL is responsible for the formation of nanoparticles and the encapsulation of DOD. The molecular weight of PCL is a critical factor, as it influences the stability, degradation rate, and efficacy of the drug-loaded nanoparticles. A higher molecular-weight PCL creates a denser, more stable matrix that minimizes premature drug release. According to the literature, the optimal molecular weight of PCL for nanoparticles most commonly ranges from 10,000 to 80,000 [54,55,56,57].

The positive effect of higher molecular weight on encapsulation is supported by the previous work with mPEG-*b*-PLA, which showed increased cytostatic drug encapsulation efficiency with greater PLA chain length [52]. Specifically, a marked improvement in encapsulation efficiency was observed upon increasing the mPEG-*b*-PLA molecular weight from 10,000 to 36,000 (at constant *M_n_* of mPEG equal to 5000). However, subsequent increase in molecular weight did not contribute to an improvement in drug loading. As a second example, the encapsulation of hydrophobic paclitaxel into PCL nanoparticles was enhanced by increasing the polymer’s molecular weight from 14,000 to 80,000 [55]. Based on these data, mPEG-*b*-PCL copolymers with an intermediate molecular weight (*M_w_* = 15,000–50,000) were synthesized and selected candidates were subsequently evaluated for their ability to encapsulate and release DOD.

Synthesis of mPEG-*b*-PCL was carried out by ring-opening polymerization of CL with the use of mPEG (*M_n_* = 5000) as the macroinitiator. To produce copolymers with distinct molecular-weight characteristics, key polymerization parameters including temperature, duration, CL-to-mPEG and CL-to-Sn(Oct)_2_ ratios were systematically varied. The details on the synthesis conditions as well as molecular weight characteristics determined by SEC for the resulting copolymers are summarized in Table 2. The representative ^1^H NMR spectrum and SEC profile of the mPEG-*b*-PCL diblock-copolymer are shown in Figure S1 (Supplementary Materials) for sample #8.

Based on the obtained data, the following trends in the variation in the copolymer’s molecular weight depending on the synthesis conditions can be observed. An increase in the reaction temperature from 120 to 135 °C (samples ##1–3, #7 and #8, Table 2), under otherwise equal conditions, contributes to an increase in the length of the hydrophobic block of PCL keeping dispersity low. The polymerization time promotes the increase in the yield of the reaction with a low influence on the polymer length under otherwise equal conditions (samples #6 and #7, #3 and #9, #8 and #10, Table 2). However, the most significant parameter, which had the greatest effect on the copolymer molecular weight, is the monomer-to-macroinitiator molar ratio ([CL]/[mPEG]) (samples #3 and #6, #10 and #11, Table 2).

Given that samples 4–6 had relatively low molecular weights and low yields, while samples #1,2 and #8 showed values quite close to each other, samples #8 (*M_w_* = 18,600), #9 (*M_w_* = 27,600) and #11 (*M_w_* = 42,500)—all obtained with high yield—were selected for further study of drug loading.

### 3.2. Computational Study of Possible Drug–Polymer Interactions

The primary driving force for encapsulating DOD into PEG-*b*-PCL nanoparticles is the non-covalent interaction between the drug and the core-forming PCL block. To clarify the molecular binding mechanism between DOD and PCL, an extensive computational analysis was performed. This analysis used a model system comprising DOD and four *ɛ*-caprolactone monomer units. A chain length of four units was selected after testing models of 1–6 units, as it provided statistically significant coverage of DOD with reasonable computational cost.

**Figure 2:**
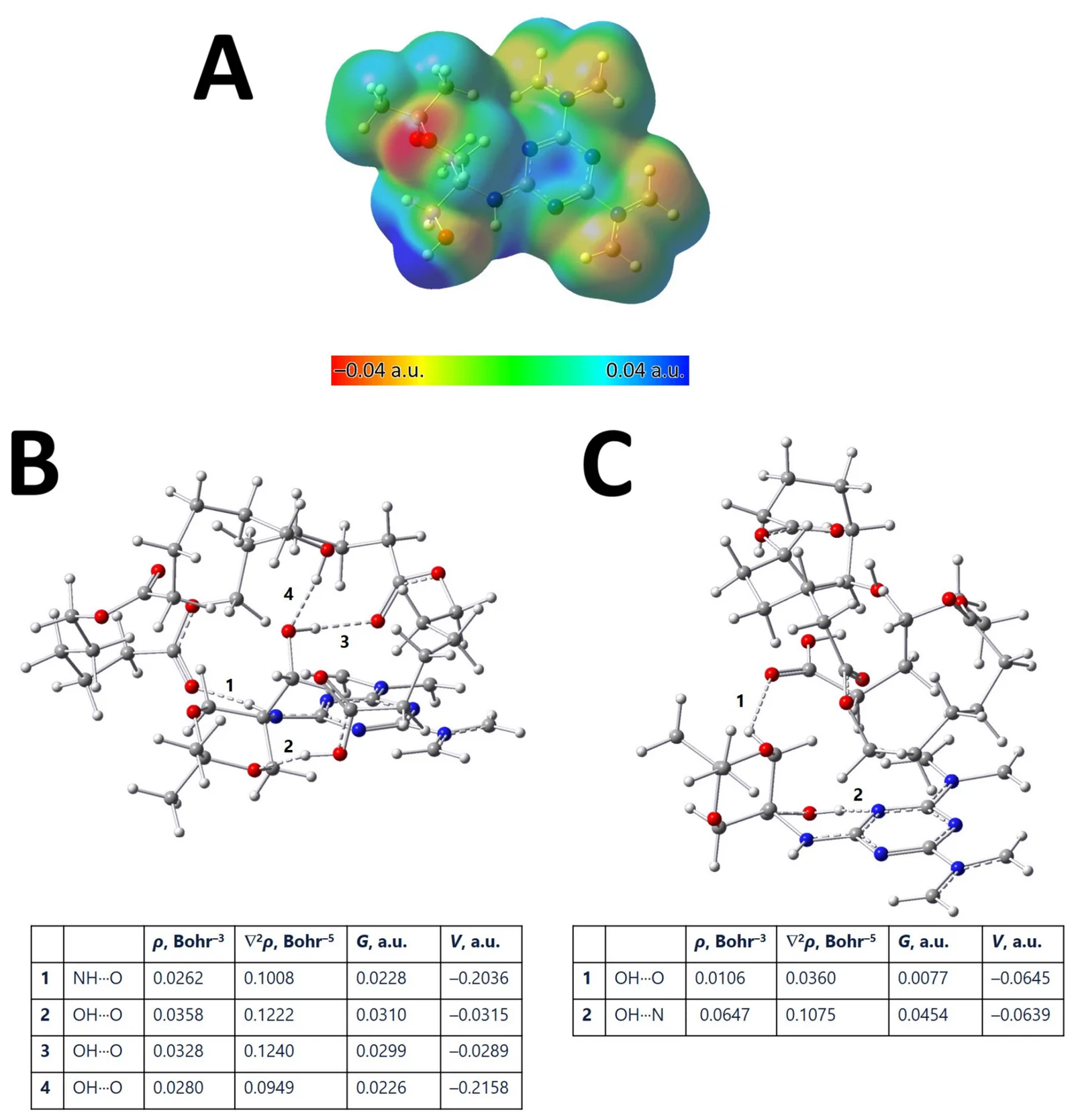
Computational analysis of DOD and PCL interactions: (**A**) Electrostatic potential distribution at the 0.001 a.u. electron density isosurface for DOD, visualized by color mapping. The most positive region (blue) corresponds to the hydroxyl proton of DOD, while the most negative region (red) is located at the dioxane ring oxygens; (**B**,**C**) Optimized structures of DOD + four-unit PCL clusters illustrating distinct hydrogen bonding assembly. Hydrogen bonds exceeding thermal energy at room temperature are indicated by dashed lines; the associated quantum topological analysis values are given in tables above. The tables above summarize key quantum topological parameters at the (3–1) critical points of electron density, including electron density *ρ*, Laplacian of electron density ∇^2^*ρ*, local kinetic electron energy density *G*, and local potential electron energy density *V*. The bond numbering corresponds to that in the structural diagrams above. (**B**) reflects the primary structure, while (**C**) demonstrates the second representative structure. The molecular structures and graphical representations were prepared using GaussView (version 6.1).

Electrostatic potential mapping was carried out at the 0.001 a.u. electron density isosurface (Figure 2A), demonstrating that the most positive region (blue) is concentrated at the hydroxyl proton of DOD, whereas the most negative (red) zone localizes at the dioxane ring oxygens. Given the excess of carbonyl moieties in PCL functioning as proton acceptors, these data suggest that hydrogen bonding between the hydroxyl proton of DOD and the PCL carbonyl is highly probable.

Conformational analysis employing the GOAT algorithm was performed for the model cluster, followed by geometry optimization for the five lowest-energy conformers at the B3LYP/6-311++G(d,p) level with Grimme’s D3BJ dispersion correction. Of these, two representative aggregated states are presented in Figure 2B,C, revealing distinct coordination patterns. For the primary complex (Figure 2B), the polymer chiefly coordinates to the DOD via the hydroxyl group, congruent with the electrostatic potential distribution. The estimated binding strength based on the local kinetic energy density *G* at the hydrogen bond critical point, using the relation *E*_HB_ = 0.429·*G* [58], yields a value of approximately 8 kcal/mol, notably exceeding kT at room temperature. In addition, a relatively strong NH···O hydrogen bond is revealed (marked as 1 in Figure 2B,C), with an estimated strength near 6 kcal/mol. Hydrogen bonds 2 and 4 originate from the terminal polymer fragment and are not discussed further, as their formation is unlikely in realistic, long-chain PCL.

**Figure 3:**
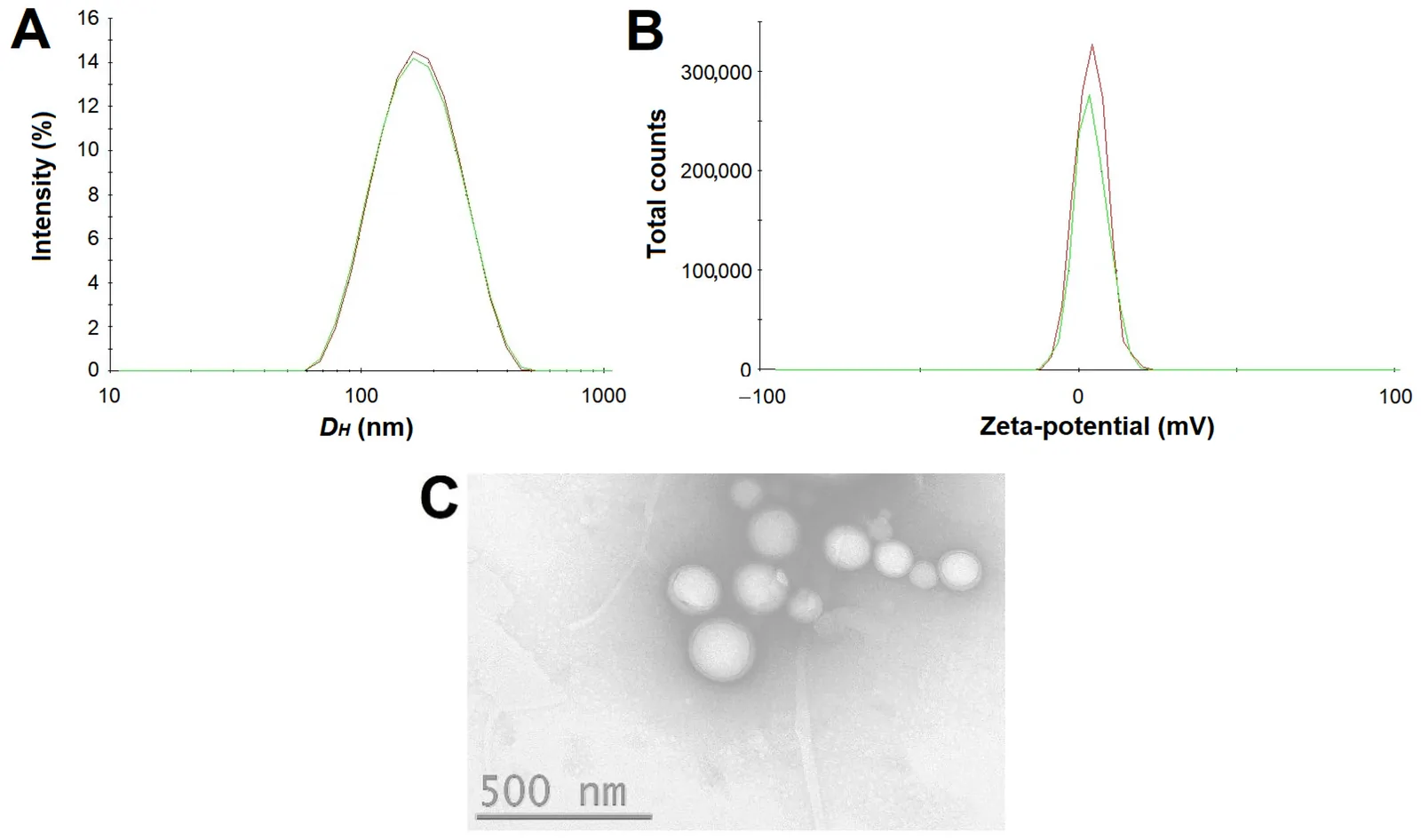
Particle size distribution (**A**), zeta-potential (**B**) (results for two measurements) and TEM image (**C**) of the mPEG-*b*-PCL@DOD nanoformulation (drug loading: 463 μg DOD/mg of polymer). The results shown herein were obtained using mPEG-*b*-PCL polymer sample with *M_w_* = 27,600 (sample #9, Table 1). The plots of different colors in (**A**,**B**) represent parallel measurements of the same sample.

The second representative structure (Figure 3C) demonstrates an alternative scenario, where the hydroxyl group of DOD forms an intramolecular hydrogen bond with the triazine nitrogen. In this case, the polymer is deprived of effective binding sites at DOD, allowing only for weak van der Waals-like contacts. The IRI (Interaction Region Indicator) maps (isosurface value 1.0), shown in Figure S2 (Supplementary Materials), allow visualization of all non-covalent interactions in model systems. Less representative conformers exhibit either scenario: (*i*) with strong carboxyl-hydroxyl hydrogen bonding, (*ii*) no effective binding, or (*iii*) marginally relevant processes where terminal polymer protons migrate to DOD, forming improbable N(CH_2_)CH_3_ groups—requiring significant activation barriers.

Overall, these computational insights corroborate the experimentally inferred predominant role of the hydroxyl group in mediating hydrogen bonding with the PCL polymer, thereby elucidating the fundamental molecular determinants of DOD encapsulation and interaction with the polymer matrix. However, the electrostatic potential distribution shows that the DOD molecule contains both polar (hydroxyl group, triazine ring) and neutral aliphatic fragments (methyl and methylene groups) (Figure 3A). The latter do not participate in electrostatic interactions but can engage in hydrophobic contacts with similar neutral regions of PCL (methylene -CH_2_- groups). Although the simulations focused primarily on hydrogen bonding (since short oligomer chains were considered), the proximity of neutral (aliphatic) fragments of DOD and PCL in the optimized structures (Figure 3B,C) and the presence of weak van der Waals contacts in the IRI maps (Figure S2, Supplementary Materials) indirectly confirm the possibility of such interactions. Thus, hydrophobic interactions complement hydrogen bonding, particularly for elongated PCL chains where the cooperative effect of hydrophobic contacts becomes significant. This understanding provides a robust foundation for interpreting the drug-polymer compatibility and guides the rational design of optimized nanoformulations discussed in subsequent sections.

### 3.3. Production and Characterization of Nanoformulations

According to the literature, drug-loaded PEG-*b*-PCL nanoparticles can be formulated within a size range of 30 to 400 nm by adjusting the preparation method [43]. However, drug encapsulation efficacy (*EE*) is predominantly influenced by the nature of the drug. A strong correlation is observed between drug hydrophobicity and *EE*; hydrophilic drugs exhibit the lowest encapsulation (as low as 3%), while more hydrophobic drugs achieve *EE* values as high as 95% [43].

In our case, the mPEG-*b*-PCL@DOD nanoformulations were prepared using the nanoprecipitation technique. This involved the dropwise addition of the polymer or polymer/DOD solution in an organic phase into water under intensive stirring. The initial polymer/DOD ratios, nanoformulation yield, drug loading, encapsulation efficacy, and characteristics of the resulting nanoformulations are summarized in Table 3. Representative size distribution and zeta-potential profiles for the mPEG-*b*-PCL@DOD nanoformulation with the highest drug loading are shown in Figure 3A,B, respectively.

The results indicate that DOD encapsulation and an increase in the initial drug content in the organic phase led to only minor changes in the hydrodynamic diameter and polydispersity index (PDI) of the resulting nanoformulations. In all cases, the hydrodynamic size of the drug-loaded nanoformulations was larger than that of the empty nanoparticles and increased slightly with higher drug loading (Table 3). All mPEG-*b*-PCL@DOD nanoformulations exhibited a hydrodynamic diameter in the range of 140–160 nm with PDI values below 0.17 confirming the formation of narrowly dispersed nanosystems. At the same time, the zeta-potential values of the nanoformulations increased upon the introduction of DOD and with an increase in its content. This phenomenon is explained by the compensation of the polymer’s negative charge by the positive charge of the drug, which is an amphiphilic base.

The characteristics of the obtained nanoformulations are consistent with those reported for similar systems. For instance, PEG-*b*-PCL nanoparticles loaded with doxorubicin, mucin1, or a combination of both drugs exhibit a comparable hydrodynamic diameter range of 100–175 nm [59]. Meanwhile, the encapsulation efficiency for the amphiphilic substance doxorubicin was 85%, a value that is close to that obtained in this study for the amphiphilic compound DOD.

TEM analysis of the mPEG-*b*-PCL@DOD nanoformulation revealed the formation of spherical nanoparticles (Figure 3C). The average particle diameter measured by TEM was 125 ± 31 nm, which is consistent with the hydrodynamic diameter of 155 nm determined by DLS. The observed ~20% size difference is typical and can be attributed to the dehydration and collapse of the hydrated PEG corona during TEM sample preparation (drying on the grid). At the same time, this hydrated corona is a key component of the hydrodynamic diameter measured by DLS.

Despite the aforementioned trends, where PLA length increases DOD encapsulation [52] and PCL length increases paclitaxel encapsulation [55], this study did not reveal a drastic difference between the mPEG-*b*-PCL samples with molecular weights (*M_w_*) in the range of 18,600–42,500. Encapsulation performed using the same initial DOD content (theoretical drug loading of 400 μg/mg of polymer) resulted in higher drug loading, nanoformulation yield, and encapsulation efficacy for mPEG-*b*-PCL with *M_w_* = 18,600 (392 μg/mg, 93%, and 91%, respectively). A decrease in drug loading, nanoformulation yield, and encapsulation efficacy was observed as the PCL block elongation, with the lowest values obtained for the copolymer with *M_w_* = 42,500 (343 μg/mg, 85%, and 73%, respectively). The reduction in nanoparticle and nanoformulation yield with the elongation of the hydrophobic block may be attributed to the lower solubility of the more hydrophobic polymer, which leads to its faster precipitation from the organic phase into the water. As a result, some polymer is lost due to uncontrolled precipitation, rather than being incorporated into the nanoparticles through nanoprecipitation. In turn, the observed slight decrease in drug loading upon elongation of the hydrophobic block may be attributed to a reduction in the number of PCL chains per fixed polymer mass, and consequently, in the number of terminal hydroxyl groups. At the same time, according to computational analysis, these groups play an important role in drug binding. It also should be noted that a longer PCL block enlarges the hydrophobic core, which, for a given mPEG length, reduces the surface density of the hydrophilic corona. This effect, in turn, weakens “stealth effect” against plasma proteins, potentially shortening circulation time of the nanomedicine [60]. Therefore, while a longer PCL block may enhance core stability and lower CMC [61], it simultaneously compromises the stealth properties conferred by PEG.

Nevertheless, characterization revealed that all obtained nanoformulations were optimal for intravenous administration, with a hydrodynamic diameter (*D_H_*) below 200 nm and a PDI below 0.2. These values meet the reported optimal size range (10–200 nm) and homogeneity (PDI < 0.3) [62], satisfying the most stringent requirements for rigid nanoparticle delivery vehicles [63].

### 3.4. DOD Release Study

**Figure 4:**
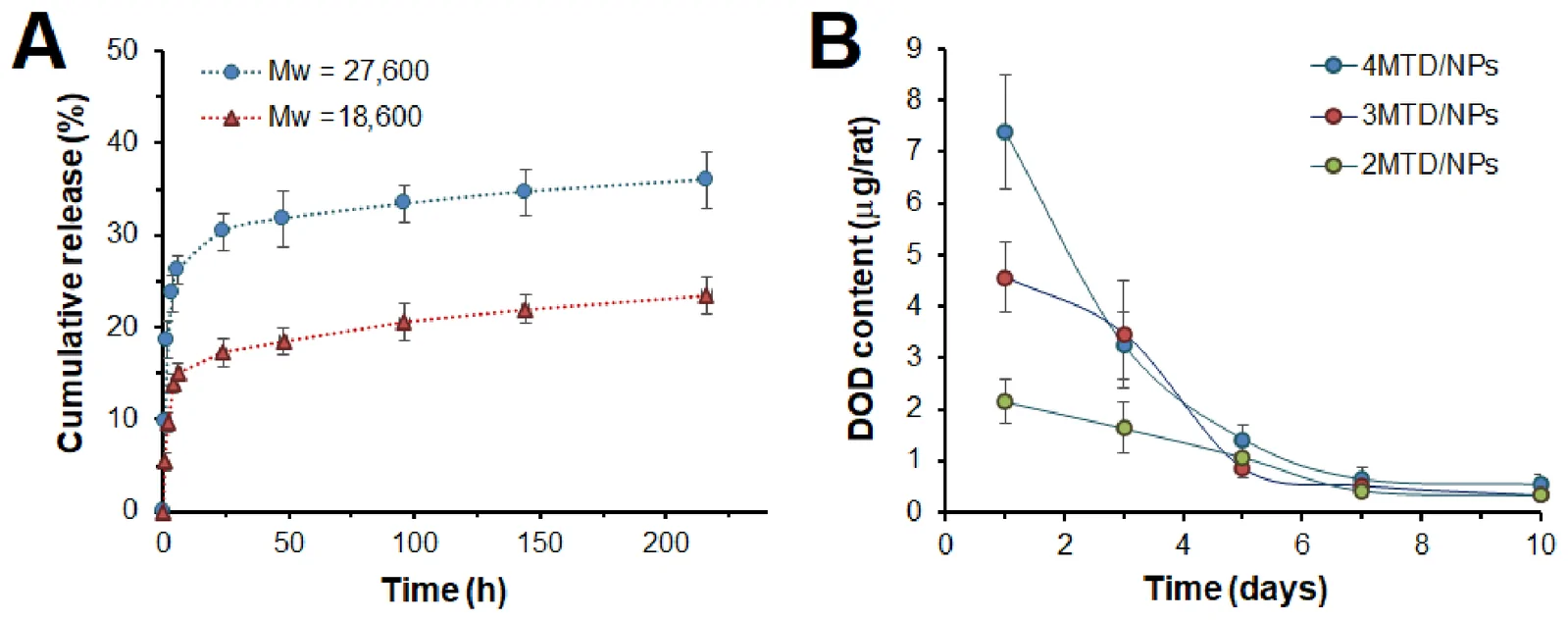
Profiles of DOD release from mPEG-*b*-PCL@DOD nanoformulations (*n* = 3): (**A**) in 0.01 M PBS (pH 7.4) for nanoformulations prepared using copolymers of different molecular weights (drug loading = 374 µg/mg of polymer, *M_w_* = 27,600; drug loading = 392 µg/mg of polymer, *M_w_* = 18,600) (**B**) in rat blood from the nanoformulation prepared with *M_w_* = 27,600 copolymer (drug loading: 374 µg/mg of polymer) depending on the dose.

In order to study DOD release, mPEG-*b*-PCL@DOD nanoformulations prepared from copolymers with *M_w_* = 18,600 and 27,600 were subjected to a dialysis method in 0.01 M PBS (pH 7.4) as the release medium at 37 °C. Figure S3 (Supplementary Materials) shows the free drug release profile. It is demonstrated that DOD is completely (99%) released from the dialysis bag within 6 days. As shown in Figure 4A, the cumulative release profile demonstrates an initial burst release of 33% and 18% of DOD within 48 h from nanoformulations based on mPEG-*b*-PCL with *M_w_* = 27,600 and 18,600, respectively. Further incubation reveled a more gradual release after 2 days, reaching 36% and 24% of DOD for the higher and lower molecular weight copolymers, respectively, over 9 days. According to computational analysis, the retention of the amphiphilic DOD molecules within the PCL matrix is governed by a combination of hydrogen bonding and hydrophobic interactions. However, DOD retention in nanoparticles based on the longer polymer was less effective, suggesting that the length of the hydrophobic PCL block is not a critical factor within the studied molecular weight range. At the same time, the use of shorter polymer chains resulted in a higher number of chains per fixed polymer mass, and consequently, a greater number of terminal hydroxyl groups in the system. These hydroxyls directly enhance hydrogen bonding with DOD leading to better drug retention.

Overall, the release rate and biphasic profile observed in this study are consistent with published results for systems based on similar polymers or containing a drug of analogous chemical structure. For instance, Piazza et al. observed a comparable pattern in a study on mPEG-*b*-PLA@methotrexate nanoformulations [64]. The authors reported that 16% to 40% of the amphiphilic anticancer drug methotrexate was released from mPEG-*b*-PLA nanoparticles within 24 h, depending on the copolymer’s composition and molecular weight. Behl et al. studied the release of the amphiphilic drug doxorubicin from ~100 nm PEG-*b*-PCL nanoparticles based on a copolymer with *M_w_* = 30,000 [59]. The observed 30% drug release within 48 h is similar to our result for DOD-loaded mPEG-*b*-PCL nanoparticles prepared from copolymer with *M_w_* = 27,600 (33% of DOD release for 48 h). As for release of 1,3,5-triazine derivatives, Chalermnon et al. detected a 30% release of the drug from calcium citrate nanoparticles after 24 h and 40% after 48 h in a buffer medium (pH 7.4) [65].

In order to evaluate the mechanism of the DOD release from the mPEG-*b*-PCL nanoparticles, an analysis of the drug release profile using a set of 10 mathematical models was performed (Table 4 and Figure S4 of Supplementary Materials). It was found that the dominant release mechanism of DOD from the mPEG-*b*-PCL nanoparticles is diffusion through a heterogeneous polymer matrix. In particular, analyzing the Korsmeyer-Peppas model (R^2^ = 0.9191), it can be concluded that the high value of the constant *k_KP_* = 17.243 indicates the presence of burst release [66], while the exponent *n* = 0.147 indicates a pronounced predominance of the polymer chain relaxation rate over the rate of water penetration into the polymer nanoparticle, which corresponds to a quasi-Fickian diffusion mechanism [67].

Moreover, the Fickian diffusion constant (*k*_1_ = 17.404) in the Peppas-Sahlin model (R^2^ = 0.9545) significantly exceeds the polymer relaxation constant (*k*_2_ = −2.146) in absolute value. This result indicates the predominance of the diffusional contribution over the processes of swelling or relaxation of polymer chains. The best mathematical description of the experimental data was achieved using the empirical Weibull model, which showed the highest coefficient of determination (R^2^ = 0.9919). As was shown by Papadopoulou and co-authors [68], values of *β* < 0.75 correspond to the Fickian diffusion mechanism in a fractal medium, which fully correlates with the obtained value of *β* = 0.125 and the conclusions based on the Peppas-Sahlin and Korsmeyer-Peppas models. It is also worth noting that the polymer does not undergo significant erosion over 216 h, since the models describing the process via dissolution or erosion of the polymer matrix (the Hixson-Crowell model (R^2^ = 0.7463) and the Hopfenberg model (R^2^ = 0.7578)) showed the lowest correlation.

According to previous studies, free DOD is rapidly eliminated from the bloodstream, with a half-life of 220 min [6]. The concentration and percentage of the initial dose represented by DOD are summarized in Table 5. Based on the previously published data [69], blood levels of free DOD account for 3.5–4.5% of the administered dose after 24 h. In turn, the percentage of DOD in the blood when using mPEG-*b*-PCL@DOD nanoformulation was 1.1–1.9% of the administered dose. If we assume that the clearance rate of DOD released from the nanoparticles is similar to that of the free substance, the observed residual DOD content in the blood for the nanoformulations corresponds to 31–43% of the free drug content. These data indirectly suggest that the in vivo drug release after 24 h amounts to approximately 31–43%, depending on the administered dose. As mentioned above, the release of DOD from the nanoparticles in a buffer medium over the same period was 30% at high DOD loading. Thus, the obtained values generally correlate with each other.

A previous study on the pharmacokinetics of DOD in rat blood showed that after 2–3 days, only trace amounts of the drug were detected [69]. In the case of mPEG-*b*-PCL@DOD nanoformulations, the blood concentrations of DOD changed over 5 days, reaching a declining plateau by day 7 (Figure 4B). Moreover, higher dosing of the mPEG-*b*-PCL@DOD nanoformulation correlated with increased drug levels in the blood. After 5 days, this dose-dependent effect was no longer evident.

### 3.5. In Vitro Biological Evaluation

#### 3.5.1. Cytotoxicity of Nanoparticles and Inhibition Activity of DOD Nanoformulation

**Figure 5:**
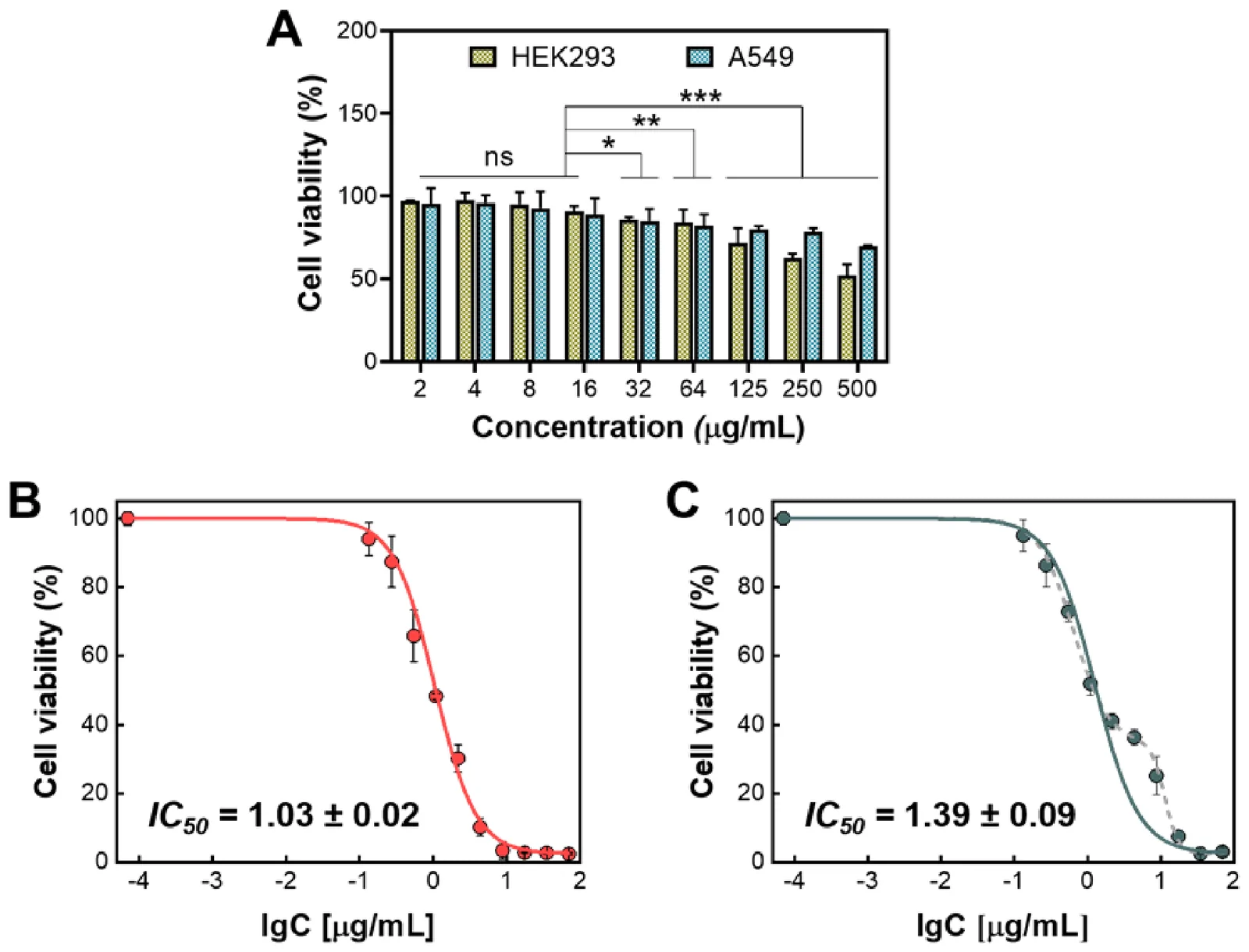
Cytotoxicity of mPEG-*b*-PCL (*M_w_* = 27,600) nanoparticles in HEK 293 and A549 cells (**A**), and the dose–response curves plotted for the inhibitory activity of free DOD (**B**) and mPEG-*b*-PCL@DOD nanoformulation (drug loading = 374 µg/mg of polymer, *M_w_* = 27,600) (**C**) in A549 cells (72 h). Data are expressed as averages of three independent experiments ± SD (*ns*, not significant; * *p* ≤ 0.05; ** *p* ≤ 0.01; *** *p* ≤ 0.001).

For in vitro biological evaluation, nanoparticles based on mPEG-*b*-PCL with *M_w_* = 27,600 were selected as the longer hydrophobic block may result in more pronounced cytotoxicity [52,70]. The cytotoxicity of the empty mPEG-*b*-PCL nanoparticles was investigated by incubating cells with corresponding objects for 72 h, followed by the MTT assay. Figure 5A illustrates the viability of normal (HEK 293) and cancer (A549) cells as a function of the concentration of the drug-free nanoparticles. For HEK 293 cells, viability remained above 70% in the concentration range of 2–125 µg/mL and above 50% at all higher concentrations tested. Conversely, A549 cells treated with the empty nanoparticles showed no cytotoxic effect across the entire concentration range, with viability remaining at 70% even at the highest concentration of 500 µg/mL. According to ISO 10993-5 criteria, a reduction in cell viability of more than 30% is considered a cytotoxic effect [71,72]. Thus, the tested empty nanoparticles were non-toxic to normal cells at concentrations up to 125 µg/mL and to cancer cells at concentrations up to 500 µg/mL, demonstrating a clear phenotype-specific effect.

The absence of cytotoxicity in other cancer cell lines at high concentrations has also been reported. For example, Su et al. demonstrated that mPEG-*b*-PCL nanoparticles were nontoxic to chondrosarcoma cells (SW1353 cell line) up to 625 µg/mL [73]. Regarding HEK 293 cells, Wang et al. reported that mPEG-*b*-PCL micelles showed no cytotoxicity at concentrations up to 1000 µg/mL [74]. However, the block copolymer used in that study had *M_n_* = 4000 with the PEG block having *M_n_* = 2000. Consequently, the molecular weight of the mPEG-*b*-PCL reported in the literature was significantly lower than that used in the present work.

The inhibitory activity of free DOD (Figure 5B) and mPEG-*b*-PCL@DOD nanoformulation was evaluated in the dose–response study using A549 cells (Figure 5C). It can be seen that the sigmoidal curve for the nanoformulation exhibits a less steep decline in cell viability (Figure 5B,C), which is a direct result of its time-dependent drug release profile. Moreover, the dose–response curve for the nanoformulation exhibits a point of inflection at a cell viability of 30–40%. This trend can be explained by the drug release kinetics of DOD (Figure 4A) showing an initial burst release of the cytostatic agent occurring within the first day, followed by a further sustained release. Due to its sustained release profile, the nanoformulation provides a lower released drug concentration over time than the free drug, which is immediately bioavailable. Nevertheless, encapsulation preserves DOD’s activity, and the nanoformulation demonstrates inhibitory efficacy comparable to that of the free drug in A549 cell culture after 72 h of coincubation. Calculation from the dose–response curves yielded *IC*_50_ values of 3.19 ± 0.09 µM for free DOD and 4.32 ± 0.27 µM for DOD in the mPEG-*b*-PCL@DOD nanoformulation (or 1.03 ± 0.02 µg/mL and 1.39 ± 0.09 µg/mL, respectively). The obtained value for free DOD is close to a recent result obtained by another research group, who determined the *IC*_50_ value to be 13.7 µM in A549 cells [8].

#### 3.5.2. Cell Cycle Analysis

**Figure 6:**
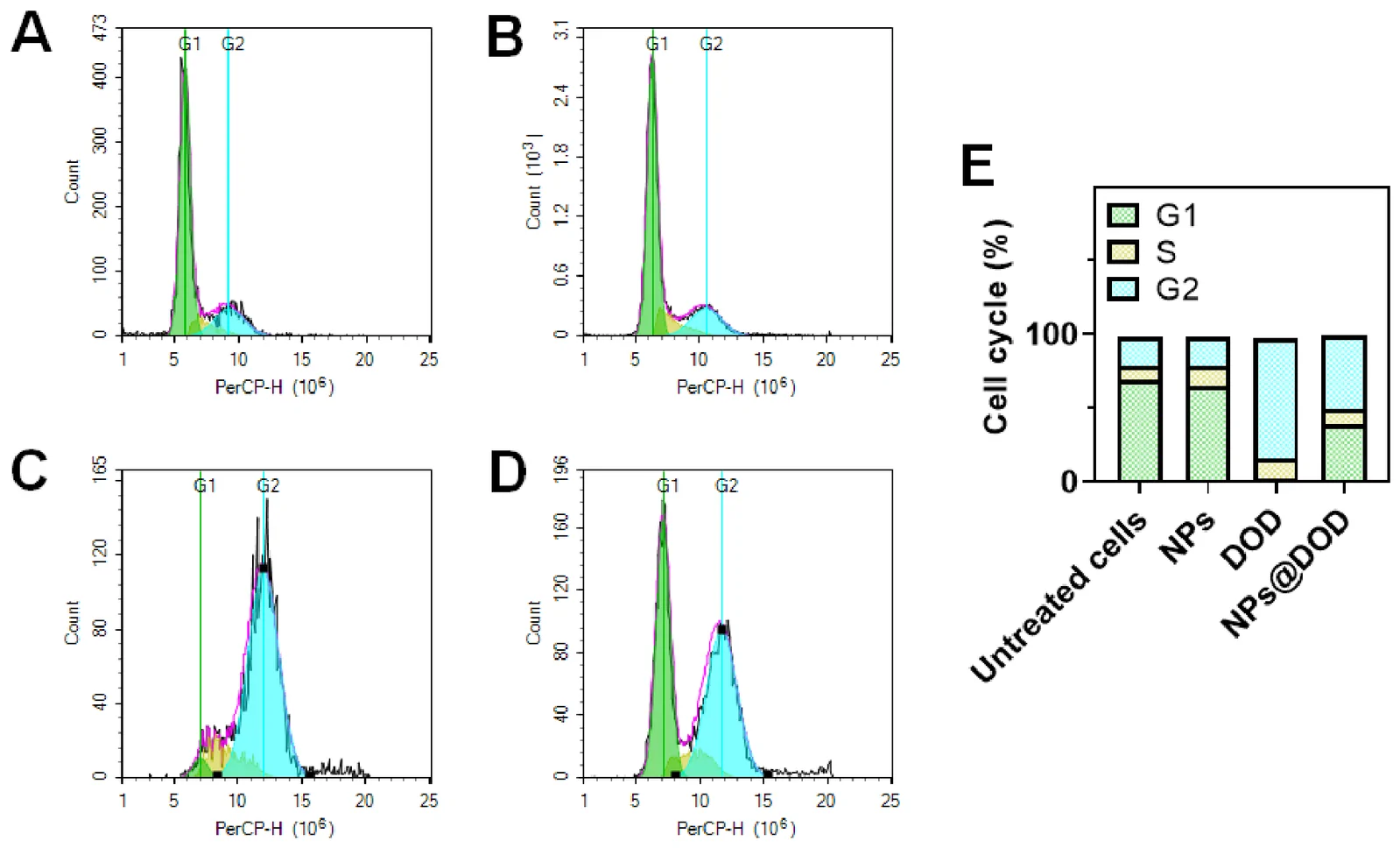
Cell cycle analysis of A549 cells: (**A**) Untreated control cells; (**B**–**D**) Cells treated with empty nanoparticles (**B**), free DOD (**C**), and the mPEG-*b*-PCL@DOD nanoformulation (drug loading = 374 µg/mg of polymer, *M_w_* = 27,600) (**D**); (**E**) Distribution of cell populations in the G1, S, and G2 phases for the results shown in panels (**A**–**D**). DOD concentration in (**C**,**D**) was the same and equal to 2 µg/mL.

The cytostatic effect of DOD and mPEG-*b*-PCL@DOD nanoformulation was further investigated through cell cycle analysis. It is known that many established drug substances with antitumor activity, such as paclitaxel [75], cisplatin [76], and doxorubicin [77], induce cell cycle arrest at the G2/M phase. According to literature data [78], DOD has a mechanism of action comparable to cisplatin. Specifically, it initiates apoptosis by inhibiting DNA synthesis, specifically via the alkylation of nucleophilic groups in nucleic acids. The results on cell cycle analysis in A549 cells revealed that both DOD and mPEG-*b*-PCL@DOD nanoformulation taken at the same DOD concentartion (2 µg/mL) indeed induce G2 arrest, demonstrating proportions of cells in the G2 phase of 80% and 50%, respectively (Figure 6). In contrast, the empty nanoparticles did not alter the cell cycle, maintaining the percentage of cells in the G1 and G2 phases at constant levels of approximately 65% for G1 and 20% for G2.

**Figure 7:**
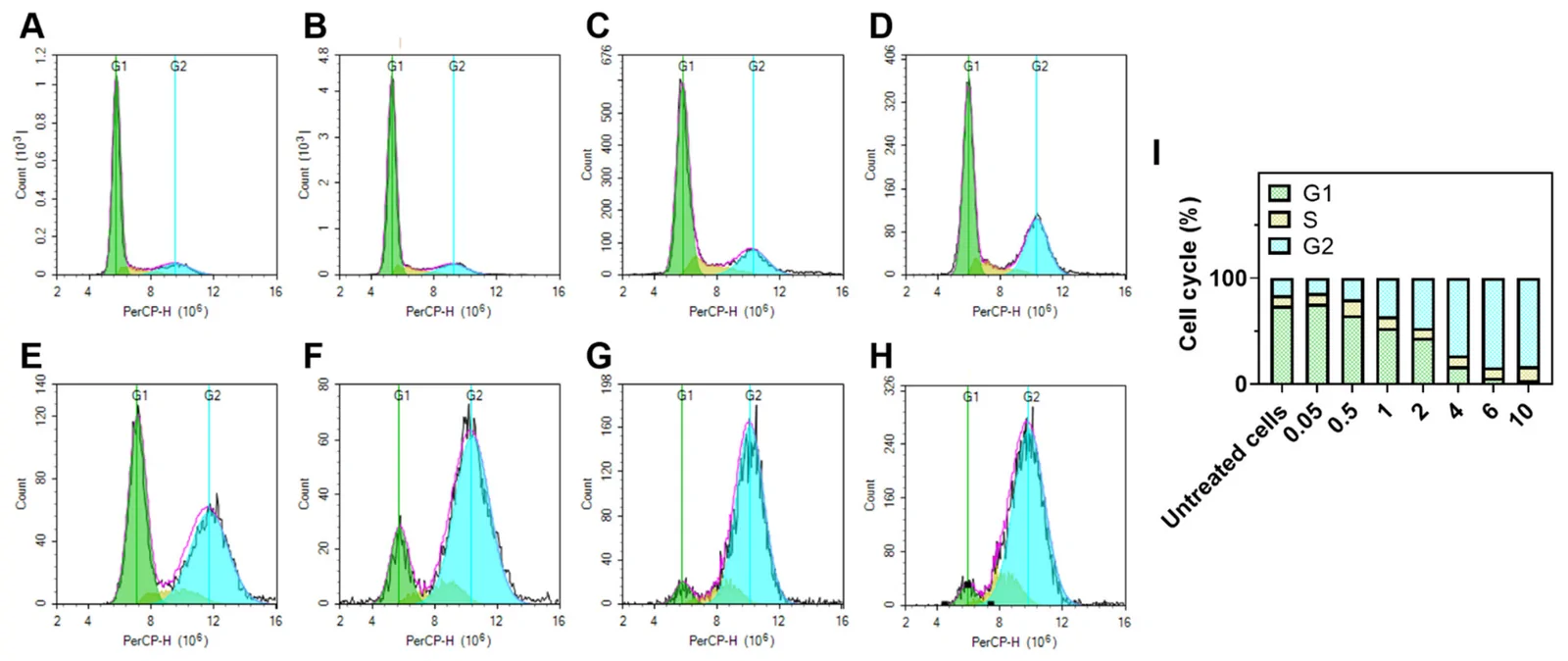
Cell cycle analysis of A549 cells treated with the mPEG-*b*-PCL@DOD nanoformulation at various DOD concentrations: (**A**) Untreated control cells; (**B**) 0.05 µg/mL; (**C**) 0.5 µg/mL; (**D**) 1 µg/mL; (**E**) 2 µg/mL; (**F**) 4 µg/mL; (**G**) 6 µg/mL; (**H**) 10 µg/mL; (**I**) Distribution of cell populations in the G1, S, and G2 phases based on the results shown in (**A**–**H**).

It should be noted that free DOD at concentrations higher than 2 µg/mL demonstrated high toxicity, resulting in an insufficient number of events to obtain a reliable cell cycle analysis. In turn, the DOD nanoformulation was tested across a concentration range of 0.05–10 µg/mL (Figure 7). The histogram in Figure 7I clearly demonstrates a dose-dependent effect. At a concentration of 10 µg/mL, the vast majority of cells are in the G2 phase, confirming the cytostatic action of the agent and its ability to block proliferation. For the DOD nanoformulation, an effect similar to that of free DOD at a concentration of 2 µg/mL (Figure 6E) was achieved at a concentration of 6 µg/mL. The significantly lower immediate toxicity can be explained by the need for drug release from the polymer nanoparticles. Consequently, this feature enables an increase in drug dosage in subsequent in vivo experiments.

### 3.6. Antitumor Activity In Vivo

It is known from the literature that the critical micelle concentration (CMC) for mPEG-*b*-PCL containing mPEG with *M_n_* = 5000 and PCL with *M_n_* = 18,300 is 0.9 mg/L, or 0.9 µg/mL [61]. The CMC value of mPEG-*b*-PCL containing the same length of mPEG but PCL with *M_n_* = 8500 is 1.8 µg/mL. In our case, the copolymer used for the in vivo study had a total *M_n_* = 20,600 (*M_w_* = 27,600; sample #9 in Table 2). Since in our case the *M_n_* value of mPEG was also 5000, the *M_n_* value of PCL was approximately 15,600. This allows us to expect a CMC value lying between 0.9 and 1.8 µg/mL.

In all doses, the injected volume of the nanoformulations was 0.2 mL. In this volume, the polymer content was 0.8, 1.2 and 1.6 mg for the 2MTD, 3MTD and 4MTD dose, respectively. Given that the mean blood volume per mouse is 1.5 mL, the polymer concentration in the blood after administration is approximately 470, 706, or 940 µg/mL for the 2MTD, 3MTD and 4MTD doses, respectively. Thus, after dilution in the blood, the polymer concentration is 2–3 orders of magnitude higher than the CMC values known for the amphiphilic copolymer employed. This indicates that, at the concentrations used, the polymer macromolecules remain associated into nanoparticles.

Based on the results of the cell cycle study, the biological effect of the mPEG-*b*-PCL@DOD nanoformulation was similar to that of free DOD when the nanoformulation dose was three times higher than that of free DOD (Section 3.5.2). Taking this into account, for the in vivo study, the free DOD dose corresponded to the known MTD value, namely, 5 mg/kg. In turn, for the mPEG-*b*-PCL@DOD nanoformulation, as was shown in cells, the effective therapeutic dose should be higher. In this regard, doses of 2MTD and 3MTD relative to free DOD, corresponding to 10 and 15 mg/kg, respectively, were selected for in vivo testing. To verify that the expected MTD for the nanoformulation is 15 mg/kg (three times higher than that of free DOD in the cell cycle study), a dose of 20 mg/kg for the nanoformulation (4MTD relative to the free drug) was also evaluated.

#### 3.6.1. Ascites Tumor Models: Ehrlich Ascites Tumor and L1210 Lymphoid Leukemia

Female BALB/c mice were intraperitoneally transplanted with Ehrlich carcinoma. A description of the experimental groups is summarized in Table 1. In all cases, treatment of experimental animals started 48 h after tumor grafting. Data on the effect of free DOD and DOD encapsulated in the PEG-b-PCL nanoparticles on the time of ascites onset in the abdominal cavity of BALB/c mice with Ehrlich carcinoma are shown in Table 6.

Free DOD at a MTD dose and mPEG-*b*-PCL@DOD at doses of MTD, 2 MTD, and 4 MTD delayed the development of ascites in mice. By day 8 after Ehrlich carcinoma grafting, the presence of ascitic fluid was visually and palpably detected in all 10 animals of the control group (I.1). In contrast, no signs of ascites were observed at this time point in groups I.2 and I.5 treated with free DOD at MTD dose and at 4MTD DOD nanoformulation (*p* < 0.0001; *p* = 0.0020, respectively). By day 12 after Ehrlich carcinoma grafting, ascites was present in all mice in the control group (I.1) and had begun to develop in mice across all experimental groups.

**Figure 8:**
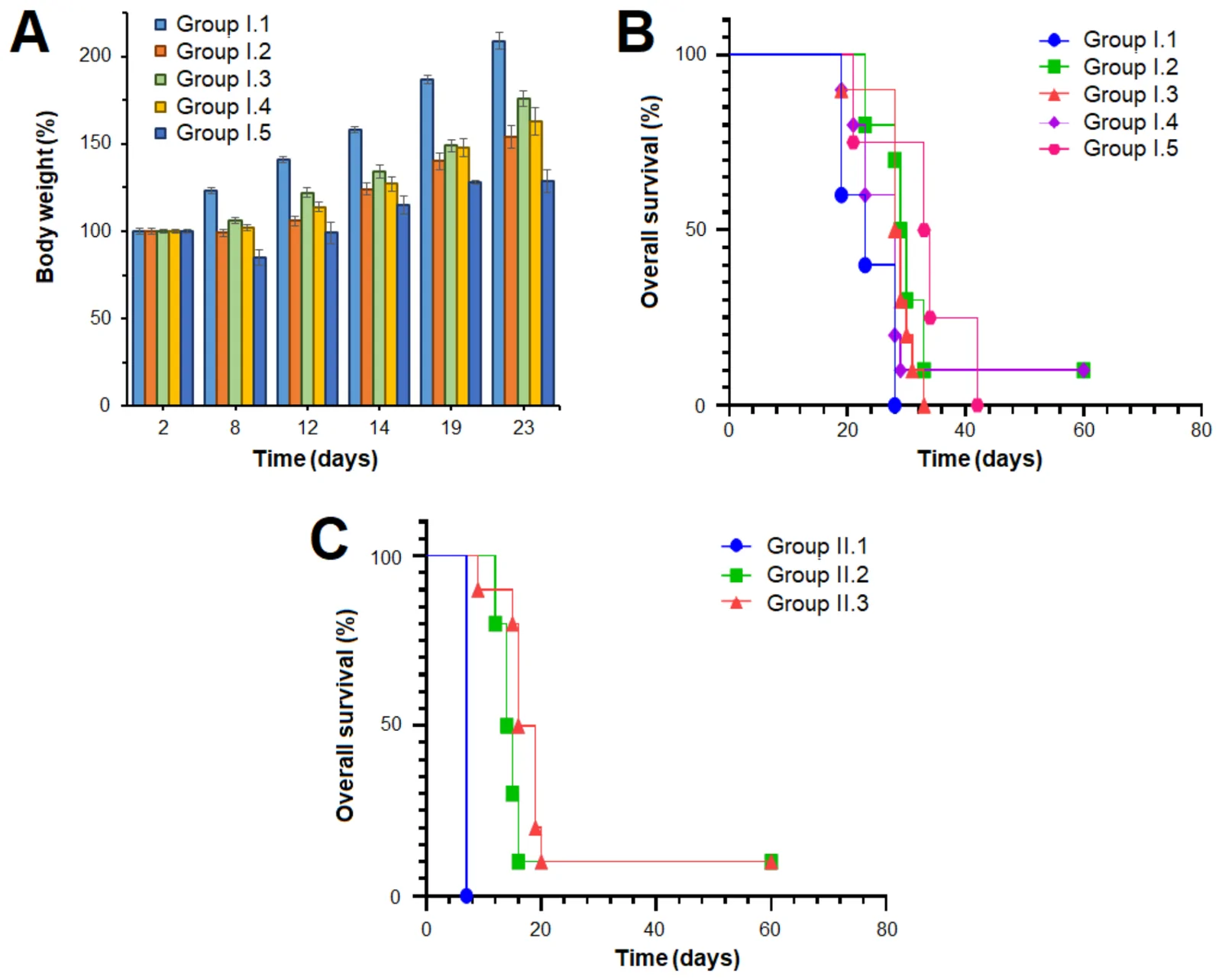
Dynamics in animal body weight (**A**), and overall survival of BALB/c mice with Ehrlich ascites carcinoma (Groups I) (**B**) and DBA mice with L1210 Lymphoid Leukemia (Groups II) (**C**). Number of animals per group was 10. Groups I.1 and II.1 were the control groups; groups I.2 and II.2 corresponded to free DOD at MTD; groups I.3-I.5 were the DOD nanoformulations at MTD, 2MTD and 4MTD; group II.3 was the DOD nanoformulation at 3MTD.

The accumulation of ascites is accompanied by an increase in animal body weight. The dynamics of body weight in mice from the control and experimental groups, and the statistical significance of these results, are summarized in Table S1 (Supplementary Materials). The percentage change in body weight over time is shown in Figure 8A. A significant increase in body weight was observed in the control group (I.1). In contrast, the increases were significantly smaller in groups I.2-I.4 received free DOD and the MTD and 2MTD DOD nanoformulations, respectively. By day 14 after tumor transplantation, a time point at which ascites had developed in all mice in the control group and most mice in the experimental groups, body weight had increased by 58% compared to baseline in the control group. In group I.2 (free DOD at MTD), group I.3 (MTD DOD nanoformulation), and group I.4 (2MTD DOD nanoformulation), the corresponding values were 24, 34, and 27%, respectively. This statistically significant difference suggests a slower development of ascites in experimental groups.

The enhanced antitumor effect observed in group I.5 (4MTD DOD nanoformulation) was accompanied by increased toxicity. This was evidenced by the premature death of animals without signs of abdominal ascites and a reduction in body weight. By day 8 after Ehrlich carcinoma transplantation, the body weight of animals in this group had decreased by 19% (*p* = 0.0286). However, body weight began to increase by day 12, a change potentially caused by the onset of ascites formation (Table S1, Supplementary Materials; Figure 8A).

Inhibition of tumor growth under the influence of free DOD at a MTD dose (group I.2) and mPEG-*b*-PCL@DOD nanoformulations at the DOD doses of MTD, 2MTD, and 4MTD (groups I.3-I.5) contributed to an increase in animal survival (Figure 8B). Free DOD (group I.2) reduced the risk of death by 88% compared to the control (group I.1) with statistical significance (HR = 0.1222; 95%CI = 0.03355–0.4450; *p* = 0.014). According to the log-rank test, administration of DOD nanoformulations at the doses of MTD and 4MTD also significantly reduced the risk of death by 86% (HR = 0.1383; 95% CI = 0.03625–0.5277; *p* = 0.0038) and 81% (HR = 0.1875; 95% CI = 0.04255–0.8262; *p* = 0.0269), respectively. A dose of 2MTD did not show a statistically significant reduction in the risk of death (HR = 0.4033; 95% CI = 0.1140–1.427; *p* = 0.1589).

The median lifespan increased with statistical significance in group I.2 (free DOD at MTD) by 28% (*p* = 0.0026), in group I.3 (MTD DOD nanoformulation) by 24% (*p* = 0.0074), and in group I.5 (4MTD DOD nanoformulation) by 46% (*p* = 0.0380) (Table 7). The increase in group I.4 (2MTD DOD nanoformulation) was not statistically significant and amounted to 22% (*p* = 0.0676). An increase in survival compared to the median survival of mice in the group I.2 was observed only for the mPEG-*b*-PCL@DOD nanoformulation at a dose of 4MTD, amounting to 13.5% without statistical significance (*p* > 0.05).

The mPEG-*b*-PCL@DOD nanoformulation at a DOD dose of MTD and 2MTD exhibited antitumor efficacy equivalent to free DOD at MTD in the Ehrlich ascites carcinoma model, but with a superior safety profile. While the 2MTD nanoformulation dose ensured 100% survival (with death later caused by tumor progression), the same dose of free DOD corresponds to LD50. mPEG-*b*-PCL@DOD nanoformulation at a DOD dose of 4MTD demonstrated both a higher antitumor effect and significant toxicity, but it was lower than that of the free substance at this dose (20 mg/kg), which reaches 100% lethal outcome. Thus, the obtained results indicate that nanoencapsulation mitigates the toxicity of DOD. This conclusion is supported by the absence of animal death at the 2MTD dose and reduced lethality at the 4MTD dose for the nanoformulations, in contrast to the high lethality of the free substance at equivalent doses.

For comparison, the high efficacy of mPEG-*b*-PCL-based delivery systems for cytostatic drugs against Ehrlich ascites carcinoma was previously demonstrated with doxorubicin. Specifically, doxorubicin-loaded mPEG-*b*-PCL micelles coated with hyaluronic acid reduced ascites volume by 2.6-fold [43].

In the second tumor ascites model, female DBA mice were grafted with L1210 lymphoid leukemia and treatment was started 48 h after tumor grafting. The median lifespan, increase in lifespan as well as overall survival are summarized in Table 8 and Figure 8C. In contrast to Ehrlich ascitic adenocarcinoma, tumor-specific mortality in the animals was 100%. The median survival in the control group (II.1) was 7 days. Treatment with free DOD at its MTD (group II.2) increased lifespan by 107% (*p* < 0.0001). In contrast, the DOD nanoformulation at 3MTD (group II.4) produced a significantly greater increase of 150% (*p* < 0.0001). Direct comparison shows the nanoformulation extended survival by an additional 21% over the free drug.

Administration of the DOD nanoformulation at 3MTD (group II.3) exhibited some toxic effect, as evidenced by a 15% decrease in mouse body weight on day 6 after tumor transplantation (day 4 after the start of treatment) compared to baseline (*p* = 0.0042) (Table S2, Supplementary Materials). However, the 3MTD dose of the encapsulated DOD, unlike the DOD substance at the same dose, was not lethal, as indicated by longer survival compared to the control and a trend towards increased survival compared to the DOD substance at MTD. Thus, in the case of the L1210 lymphoid leukemia model in DBA mice (females), DOD encapsulated into nanoparticles based on mPEG-*b*-PCL exhibited antitumor activity comparable or exceeding that of the free DOD at MTD without increasing toxicity. According to the log-rank test, treatment with both free DOD at MTD and DOD nanoformulation at a dose of 3MTD significantly reduced the risk of death in DBA mice with lymphoid leukemia L1210 by 98% (HR = 0.02237; 95% CI = 0.0040–0.1235; *p* < 0.0001).

#### 3.6.2. Solid Tumor Model: Lewis Lung Epidermoid Carcinoma

Here, female C57BL/6 mice were used for tumor grafting, and as in previous cases, treatment began 48 h after grafting. Between days 7 and 12 post-inoculation and treatment initiation, four animals in group III.4 and one animal in group III.3 died from toxicity before any tumor development was observed. Tumor growth initiated on day 24 in all animals in group III.1 (control group), in 8 out of 10 animals in group III.2 (free DOD at MTD), in 4 out of 9 animals in group III.3 (DOD nanoformulation at 3MTD), and in 3 out of 6 animals in group III.4 (DOD nanoformulation at 4MTD).

**Figure 9:**
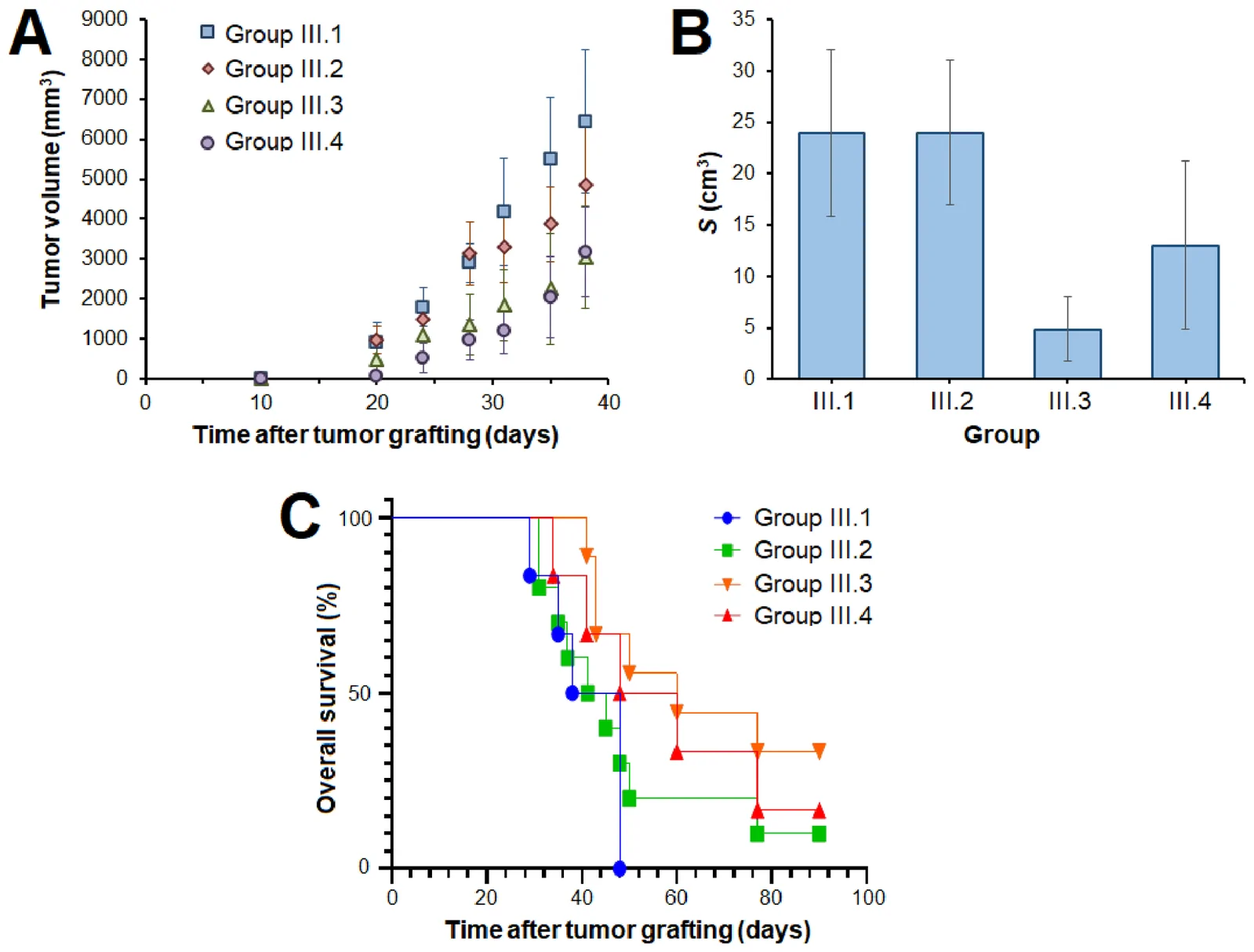
Effect of treatment with free DOD (at MTD) and mPEG-*b*-PCL@DOD nanoformulation (at 3MTD and 4MTD, relative to DOD) of the C57Bl/6 mice grafted with Lewis lung carcinoma: (**A**) Tumor volume over time following tumor grafting; (**B**) Area under the tumor growth curve (*S*) for the different experimental groups; (**C**) Overall survival over time following tumor grafting. Number of animals per group: group III.1 (control)—6 mice; group III.2 (free DOD at MTD)—10 mice; group III.3 (DOD nanoformulation at 3MTD)—9 mice; and group III.4 (DOD nanoformulation at 4MTD)—6 mice.

The tumor growth inhibition (*TGI*) of Lewis lung epidermoid carcinoma growth at different time points after tumor transplantation was 39–59% in group III.4 (DOD nanoformulation at 4MTD), and 50–92% in group III.3 (DOD nanoformulation at 3MTD). In both cases, *p* value was <0.05 regarding control group. In Group III.2 (free DOD at MTD), tumor growth inhibition was statistically significant only from day 31 to day 35 after tumor transplantation and ranged from 21–24%. Tumor volume data and *TGI* values for the different experimental animal groups and time points are summarized in Table S3 (Supplementary Materials). The growth curves of the Lewis lung epidermoid carcinoma are presented in Figure 9A. Thus, the mPEG-*b*-PCL@DOD nanoformulation exhibited pronounced inhibition of tumor growth at both the 3MTD and 4MTD doses compared to the free drug.

The key parameters for evaluating antitumor activity, which account for both the magnitude and the sustainability of the effect, are the area under the tumor growth kinetic curve (*S*) (Figure 9B) and the tumor growth index (*TG*) (Figure S2, Supplementary Materials). The minimal area under the tumor growth kinetic curve was detected to group III.3 corresponding to mPEG-*b*-PCL@DOD nanoformulation at 3MTD relative DOD. *TG* value from day 0 to day 28 post-tumor implantation was statistically significant in group III.3 with values of 20% (*p* = 0.0068), while in groups III.4 *TG* value was higher, but without statistical significance at 54%. *TG* value in group III.2 (free DOD) was 100% indicating no difference from the control group.

Overall survival is shown in Figure 9C, and the treatment’s impact on lifespan metrics (median, mean, and percentage increase) is summarized in Table 9. All animals in the control group (Group III.1) died from tumor progression within 48 days post-tumor grafting. In contrast, Group III.3 (nanoformulation at 3MTD) showed a median survival of 60 days, with 3 out of 9 mice surviving tumor-free beyond this point. This represented a statistically significant 40% increase in overall survival (Table 9).

According to the log-rank test, treatment with free DOD at the MTD reduced the risk of death from tumor progression by 25% (HR = 0.7494; 95% CI = 0.2661–2.193; *p* = 0.4025). The mPEG-*b*-PCL@DOD nanoformulation at 4MTD (relative to DOD) reduced the risk by 53% (HR = 0.4740; 95% CI = 0.1386–1.620; *p* = 0.1249); however, these reductions were not statistically significant. In contrast, the nanoformulation at 3MTD achieved a significant 84% reduction in the risk of death (HR = 0.1624; 95% CI = 0.03654–0.7215; *p* = 0.0169).

Thus, in a C57Bl/6 mouse model of Lewis lung carcinoma, the mPEG-*b*-PCL@DOD nanoformulation at a does 3MTD (relative to DOD) demonstrated a significant antitumor effect that was unachievable with free DOD at its maximum tolerated dose.

It should be noted that, for two models (leukemia L1210 and Lewis epidermoid carcinoma), a dose of 4MTD (20 mg/kg) relative to free DOD was toxic, manifested as body weight loss in the animals, whereas the 3MTD dose was generally well tolerated. Based on this, the MTD for the encapsulated mPEG-*b*-PCL@DOD nanoformulation can be presumed to be 15 mg/kg, which is three times higher than the MTD value of free DOD (5 mg/kg). This result is consistent with the results of the cell cycle study, which also showed that effective G2/M arrest for the mPEG-*b*-PCL@DOD nanoformulation was achieved at a DOD concentration three times higher than that of free DOD.

## 4. Conclusions

In this study, mPEG-*b*-PCL block copolymers of varying molecular weights were synthesized via ring-opening polymerization. By adjusting the polymerization parameters, the molecular weight of the PCL block was successfully controlled, with the monomer-to-initiator ratio appearing as the most significant factor. A number of DOD nanoformulations were prepared by nanoprecipitation, with variations in both the molecular weight (*M_w_* from 18,600 to 42,500) and the initial DOD content in the system. The resulting nanoformulations produced exhibited excellent physicochemical characteristics (size < 200 nm, PDI < 0.2) with the highest yield, drug loading and encapsulation efficacy for the copolymer with *M_w_* = 18,600. DOD release from mPEG-*b*-PCL nanoparticles followed a sustained profile, with the formulation based on the *M_w_* = 18,600 copolymer exhibiting a slower release rate. Extensive computational analysis revealed that a combination of hydrogen bonding and hydrophobic interactions between DOD and PCL governs drug encapsulation and retention within the PCL core. Overall, the developed DOD nanoformulations exhibit characteristics highly suitable for intravenous administration.

The empty nanoparticles were well-tolerated by both normal (HEK 293) and cancer (A549) cells. The nanoformulation demonstrated inhibitory activity against A549 cells with an *IC*_50_ of 4.3 µM, comparable to that of free DOD (3.2 µM). Determination of DOD concentration in blood of healthy rats indicated that the nanoformulation extended DOD’s circulation time. Furthermore, the mPEG-*b*-PCL@DOD nanoformulation shown antitumor activity in Ehrlich ascites carcinoma, L1210 lymphoid leukemia, and Lewis lung epidermoid carcinoma murine models, effectively delaying disease progression and increasing median survival. A key finding is the dramatic reduction in systemic toxicity upon DOD encapsulation. Doses of the nanoformulation that were 100% lethal in their free form (2MTD and 3MTD) were well-tolerated, allowing for the administration of higher, more effective drug doses, especially in the case of solid tumor. The study shows that encapsulating DOD into mPEG-*b*-PCL represents a promising strategy for future development of DOD nanomedicines for effective and safe cancer therapy.

Nevertheless, several limitations of this study should be addressed in future work. In particular, the study is limited to preclinical evaluation in murine models, and pharmacokinetics was assessed in healthy animals without biodistribution data. Moreover, the effect of sterilization methods on the integrity of the mPEG-*b*-PCL@DOD nanoformulations, and the scalability of the formulation remain to be determined. Therefore, despite the promising preclinical results, further studies are required before this approach can be considered for clinical translation.

## Figures and Tables

**Table 1:** Experimental groups involved in the study.

Model	Group		Treatment	DOD Dose (mg/kg)
**Ehrlich ascitic adenocarcinoma** (BALB/c mice)	I.1	Control	Sterile 0.9% NaCl solution	0
	I.2	MTD	Free DOD	5
	I.3	MTD/NPs	PEG-*b*-PCL@DOD	5
	I.4	2MTD/NPs	PEG-*b*-PCL@DOD	10
	I.5	4MTD/NPs	PEG-*b*-PCL@DOD	20
**L1210 lymphoid leukemia** (DBA mice)	II.1	Control	Sterile 0.9% NaCl solution	0
	II.2	MTD	Free DOD	5
	II.3	3MTD/NPs	PEG-*b*-PCL@DOD	15
**Lewis’s lung epidermoid carcinoma** C57Bl/6 mice	III.1	Control	Sterile 0.9% NaCl solution	0
	III.2	MTD	Free DOD	5
	III.3	3MTD/NPs	PEG-*b*-PCL@DOD	15
	III.4	4MTD/NPs	PEG-*b*-PCL@DOD	20

**Table 2:** Polymerization conditions, yields and molecular-weight characteristics of mPEG-*b*-PCL copolymers.

Sample #	Polymerization Conditions *^a^*			Polymer Yield (%)	Molecular-Weight Characteristics (SEC)		
	Temperature (°C)	Time (h)	[CL]/ [mPEG]		*M_w_*	*M_n_*	*Đ*
1	120	4	150	65	18,200	14,850	1.23
2	125	4	150	78	19,700	16,300	1.21
3	130	4	150	81	22,400	18,000	1.25
4 *^b^*	130	4	50	78	13,200	10,000	1.37
5 *^b^*	130	4	80	74	14,400	13,350	1.08
6	130	4	100	70	15,000	14,500	1.03
7	130	7	100	84	16,400	14,650	1.12
8	135	7	100	90	18,600	16,500	1.13
9	130	10	150	93	27,600	20,600	1.34
10	135	24	100	95	21,800	15,450	1.41
11	135	24	200	95	42,500	27,400	1.55

***^a^*** [CL]/[Sn(Oct)_2_] was equal to 1000; ***^b^*** [CL]/[Sn(Oct)_2_] was equal to 500.

**Table 3:** Characteristics of empty nanoparticles and DOD nanoformulations based on mPEG-*b*-PCL copolymer (obtained by nanoprecipitation technique).

Polymer/ DOD Ratio (wt/wt)	Theoretical *DL* (µg/mg of Polymer)	Yield (%)	*DL* (µg/mg of Polymer)	*EE* (%)	*D_H_* (nm)	*PDI*	Zeta- Potential (mV)
mPEG-*b*-PCL with *M_w_* = 18,600							
40/0	0	95	0	0	129 ± 7	0.09	−23.7 ± 3.9
40/16	400	93	392 ± 7	91	141 ± 14	0.11	−4.6 ± 3.3
mPEG-*b*-PCL with *M_w_* = 27,600							
40/0	0	87	0	0	140 ± 13	0.10	−22.3 ± 4.9
40/10	250	82	252 ± 8	81	152 ± 15	0.11	−10.2 ± 4.2
40/16	400	93	374 ± 11	86	155 ± 21	0.14	4.3 ± 2.7
52/26	500	81	463 ± 15	75	159 ± 19	0.12	4.0 ± 1.9
mPEG-*b*-PCL with *M_w_* = 42,500							
40/0	0	87	0	0	135 ± 23	0.17	−24.5 ± 5.3
40/10	250	69	236 ± 6	65	146 ± 12	0.14	−6.5 ± 3.7
40/16	400	85	343 ± 9	73	154 ± 19	0.13	3.4 ± 2.4

**Table 4:** Correlation coefficients and model parameters calculated for the DOD release profile from mPEG-b-PCL@DOD nanoforms.

Model	Equation	R^2^	Parameters
Zero-order	*F* = *k*_0_ × *t*	0.7230	*k*_0_ = 0.233
First-order	*F* = 100 × [1 − *Exp*(−*k*_1_ × *t*)]	0.7580	*k*_1_ = 0.003
Higuchi	*F* = *k_H_* × *t*^0.5^	0.8342	*k_H_* = 3.305
Korsmeyer-Peppas	*F* = *k_KP_* × *t^n^*	0.9191	*k_KP_* = 17.243
			*n* = 0.147
Hixson-Crowell	*F* = 100 × [1 − (1 − *k_HC_* × *t*)^3^]	0.7463	*k_HC_* = 0.001
Hopfenberg	*F* = 100 × [1 − (1 − *k_HB_* × *t*)*^n^*]	0.7578	*k_HB_* = 6.337 × 10^−6^
			*n* = 518.773
Baker-Lonsdale	3/2 × [1 − (1 − *F*/100)^2/3^] − *F*/100 = *k_BL_* × *t*	0.8469	*k_BL_* = 0.00024
Peppas-Sahlin	*F* = *k*_1_ × *t^m^* + *k*_2_ × *t*^(2×*m*)^	0.9545	*k*_1_ = 17.404
			*k*_2_ = −2.146
			*m* = 0.285
Weibull	*F* = 100 × {1 − *Exp*[−((*t* − *T_i_*)*^β^*)/*α*]}	0.9919	*α* = 4.298
			*β* = 0.125
			*T_i_* = 0.999
Gompertz	*F* = 100 × *Exp*{−*α* × *Exp*[−*β* × *log*(*t*)]}	0.9404	*α* = 1.835
			*β* = 0.270

**Table 5:** Dose-dependent DOD blood levels in healthy rats 24 h post-administration (mPEG-*b*-PCL@DOD containing 374 µg/mg of polymer, *M_w_* = 27,600).

Group	Dose (µg/Rat)	DOD Concentration (µg/mL)		% of the Initial Dose ^b^	
		Free Drug ^a^	Nanoformulation	Free Drug ^a^	Nanoformulation
2MTD	200	0.7 ± 0.1	0.15 ± 0.04	3.5	1.1
3MTD	300	–	0.30 ± 0.07	–	1.5
4MTD	400	1.8 ± 0.1	0.49 ± 0.15	4.5	1.9

**^a^** Calculated based on previously published data [69]; ^b^ Calculated according to animal weight.

**Table 6:** The onset of ascites in mice after tumor grafting and treatment.

Group		Animals Without Ascites After Tumor Grafting (%), (*p* Value Regarding Control)		
		8 Days	12 Days	14 Days
I.1	Control	0	0	0
I.2	MTD **^a^**	100 (<0.0001)	80 (0.001)	40 (>0.05)
I.3	MTD/NPs	70 (0.013)	70 (>0.05)	10 (>0.05)
I.4	2MTD/NPs	90 (0.0004)	70 (>0.05)	10 (>0.05)
I.5	4MTD/NPs	100 (0.002)	80 (0.018)	70 (0.018)

**^a^** The MTD of free DOD in mice is 5 mg/kg.

**Table 7:** Effect of treatment with different DOD formulations on lifespan of BALB/c mice with Ehrlich ascites carcinoma.

Group		Median Lifespan (Days)	Confidential Interval (95%)	Increase in Lifespan (%)	*p* Value *
I.1	Control	23	19–28	–	–
I.2	MTD	30	23–33	30	0.0026
I.3	MTD/NPs	29	28–31	26	0.0074
I.4	2MTD/NPs	28	21–29	22	0.0676
I.5	4MTD/NPs	34	21–42	48	0.0380

* Compared to group I.1 (control).

**Table 8:** Effect of treatment with different DOD formulations on lifespan of DBA mice with L1210 lymphoid leukemia.

Group		Median Lifespan (Days)	Confidential Interval (95%)	Increase in Lifespan (%)	*p* Value *
II.1	Control	7.0	7–7	–	–
II.2	MTD	14.5	12–16	107	<0.0001
II.3	3MTD/NPs	17.5	15–21	150	<0.0001

* Compared to group II.1 (control).

**Table 9:** Effect of treatment with different DOD formulations on lifespan of C57Bl/6 mice grafted with Lewis lung carcinoma.

Group		Median Lifespan (Days)	Confidential Interval (95%)	Increase in Lifespan (%)	*p* Value *
III.1	Control	43.0	29–48	–	–
III.2	MTD	43.0	31–77	0	0.6916
III.3	3MTD/NPs	60.0	43–90	40	0.0326
III.4	4MTD/NPs	54.0	34–90	26	0.1926

* Compared to group III.1 (control).

## Data Availability

Data are available within the manuscript and its Supplementary Materials.

## Supplementary Materials

The following supporting information can be downloaded at https://www.mdpi.com/article/10.3390/polym18192409/s1, Figure S1: ^1^H NMR spectrum (CDCl_3_, 25 °C) (A) and SEC trace (THF, 50 °C) (B) for mPEG-*b*-PCL diblock copolymer (sample #7 in Table 1, main text); Figure S2: IRI (Interaction Region Indicator) isosurfaces (1.0 a.u.) display non-covalent interaction zones in two representatives of DOD:PCL model aggregates. The color scale highlights interaction strengths and types; Figure S3: (A) Profile of free DOD release in 0.01 M PBS (pH 7.4) (B) The calibration plots for DOD PBS; Figure S4: (A) Zero-order; (B) First-order; (C) Higuchi; (D) Korsmeyer-Peppas; (E) Hixson-Crowell; (F) Hopfenberg; (G) Baker-Lonsdale; (H) Peppas-Sahlin; (I) Weibull; (J) Gompertz; Table S1: Dynamics in body weight of BALB/c mice after tumor grafting and treatment; Table S2: Dynamics in body weight of DBA mice with L1210 lymphoid leukemia after tumor grafting and treatment; Table S3: Dynamics in tumor growth in C57Bl/6 mice grafted with Lewis lung carcinoma.

## Abbreviations

The following abbreviations are used in this manuscript:

mPEG	Poly(ethylene glycol) monomethyl ether
PCL	Poly(*ε*-caprolactone)
PLA	Poly(lactic acid)
DOD	Dioxadet
NPs	Nanoparticles
MTD	Maximum tolerated dose
PBS	Phosphate-buffered saline
PDI	Polydispersity index
SEC	Size-exclusion chromatography
TEM	Transmission electron microscopy
NMR	Nuclear magnetic resonance
DLS	Dynamic light scattering
ELS	Electrophoretic light scattering
EE	Encapsulation efficacy
TG	Tumor growth index
TGI	Tumor growth inhibition
CI	Confidential interval
