# Supplementary material for: mPEG-b-PCL-Based Nanoformulations of Aziridine-Functionalized 1,3,5-Triazine Derivative for Cancer Treatment
Source: Polymers (Basel). 2026 Oct 2;18(19):2409. doi: 10.3390/polym18192409 (PMC13667229; doi:10.3390/polym18192409)
Supplement: Supplementary file 1 [file polymers-18-02409-s001.zip › polymers-4556498-supplementary.pdf]

mPEG-*b*-PCL-Based Nanoformulations of Aziridine-Functionalized 1,3,5-Triazine Derivative for Cancer Treatment

Irina Bagaeva, Ekaterina Sinitsyna, Yuliia Zmitrichenko, Gregorii Tochilnikov, Elena Tupikina, Sergei Silonov, Mikhail Orlov, Viktor Korzhikov-Vlakh, and Evgenia Korzhikova-Vlakh

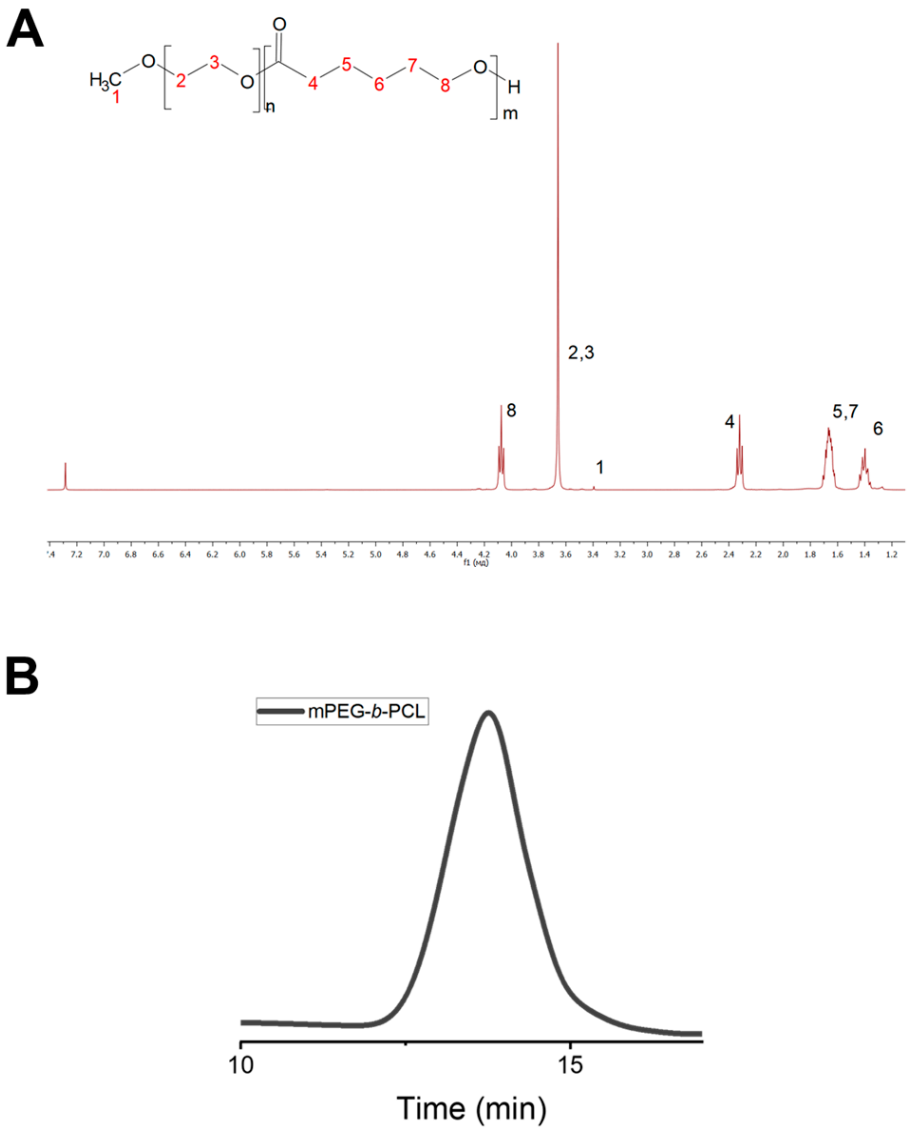

Figure S1. ^{1}H NMR spectrum ($CDCl_3$, 25 °C) **(A)** and SEC trace (THF, 50 °C) **(B)** for mPEG-*b*-PCL diblock copolymer (sample #7 in Table 1, main text).

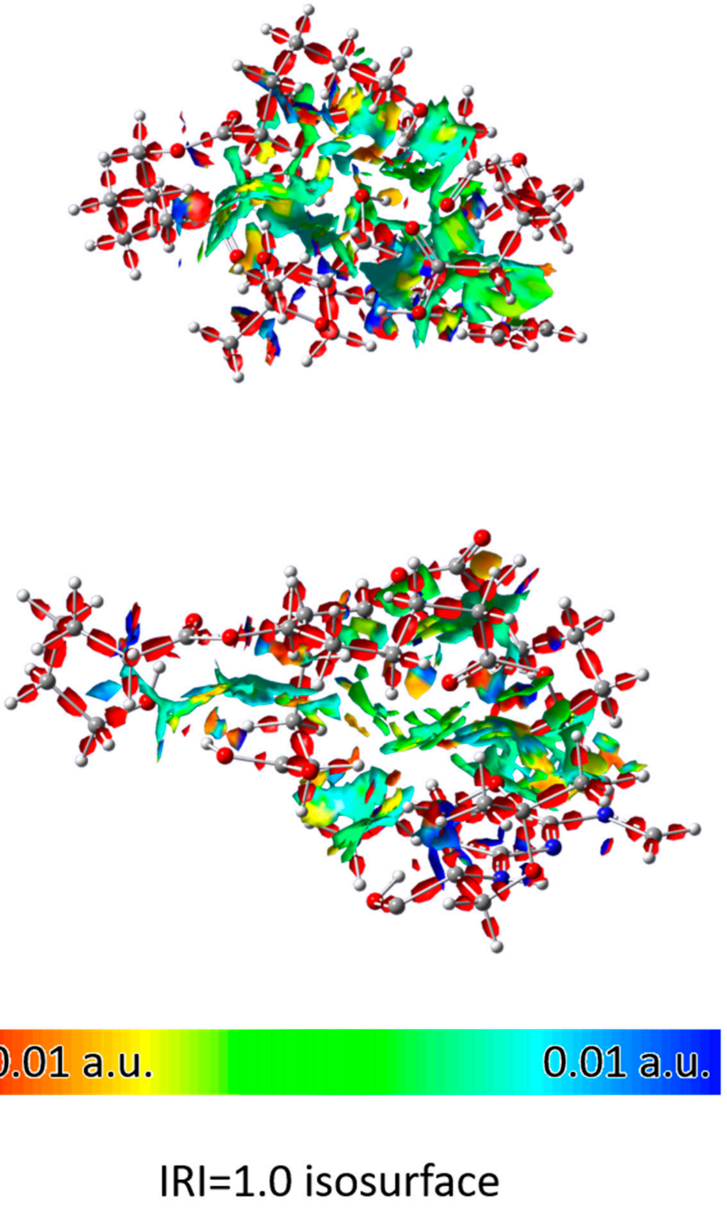

Figure S2. IRI (Interaction Region Indicator) isosurfaces (1.0 au) display non-covalent interaction zones in two representatives of DOD:PCL model aggregates. The color scale highlights interaction strengths and types.

Coordinates of equilibrated DOD:

N 0.72611400 0.61726800 -0.19623700

N 2.08313100 -1.33993200 -0.12891200

N 3.07131800 0.79565700 0.01283000

N 1.73913100 2.75002000 -0.06368800

N 4.43117500 -1.15020900 0.09234800

N -0.18903600 -1.52911300 -0.33479700

C 1.85015400 1.29772300 -0.08476000

C 0.88115100 -0.72131200 -0.21619200

C 3.11184500 -0.53494100 -0.01504400

C 4.46737800 -2.49276100 0.15137100

C 5.47855000 -0.30452700 0.12244800

C 0.67696100 3.27544900 -0.70014400

Coordinates of equilibrated DOD:PCL model complexes

1

C 1.27695600 -0.28194300 2.43168900

C 0.51120600 -0.64699500 3.67980100

H 0.96964700 -1.53934000 4.11483700

H 0.69389800 0.17673700 4.37882900

C -0.98970000 -0.82877900 3.46812000

H -1.46089700 -0.93570700 4.45188800

H -1.38855100 0.08896600 3.02856700

C -1.33817100 -2.03748200 2.59299300

H -0.95059900 -1.87716100 1.58481200

H -0.83088100 -2.92569600 2.98654700

C 2.70660100 3.40004200 0.60406700
H 0.56256300 4.34610200 -0.68765600
H -0.00259200 2.61704300 -1.20136900
H 2.66356100 4.47620600 0.62497100
H 3.50605900 2.83153000 1.03598900
H 5.29773300 0.74789500 0.04356900
H 6.46056000 -0.73924300 0.20550700
H 5.43592800 -2.95855800 0.22075500
H 3.54552100 -3.03673200 0.12607800
H 0.01831900 -2.50971400 -0.19081300
C -1.60860300 -1.17779000 -0.23462900
C -1.93024000 -0.58577200 1.15431700
C -2.03369700 -0.15998400 -1.31025700
C -3.64355800 0.79431900 0.19222400
C -5.15045000 0.94189100 0.28158800
C -2.92200600 2.13033100 0.38273700
O -3.28229400 -0.14780700 1.20465500
O -3.38474300 0.23180800 -1.09859700
O -1.85406500 -3.46294000 0.43900300
H -2.37889600 -4.26556100 0.37710500
H -1.36236400 0.69738000 -1.29430700
H -1.99672200 -0.61353200 -2.30223500
H -1.23860700 0.23142100 1.37256400
H -1.82642500 -1.35481400 1.91880000
H -1.83660900 2.04423700 0.34053600
H -3.23643200 2.82608800 -0.39721200
H -3.18957700 2.54714300 1.35519600
H -5.42789500 1.32729200 1.26340800
H -5.50347200 1.62860600 -0.48877100
H -5.61891600 -0.03170100 0.13458700
C -2.38809500 -2.47876800 -0.45167300
H -2.26883900 -2.80210600 -1.49282100
H -3.44285800 -2.28669900 -0.25149000
C -2.84143000 -2.33636300 2.49602700
H -2.99906100 -3.12893200 1.76051800
H -3.22887800 -2.69335600 3.45650500
C -3.61929900 -1.11520500 2.04553400
H -3.10742800 -0.61479500 1.22916800
H -3.77950900 -0.39029700 2.84481800
O -4.92240700 -1.54646600 1.55518200
C -5.61887000 -0.64787800 0.86577800
C -6.81567900 -1.25559300 0.17717600
H -7.05582800 -2.19469600 0.67866000
H -6.45866400 -1.53415300 -0.81887700
C -8.07673900 -0.37384000 0.10052300
H -8.46542700 -0.26318300 1.11851000
H -8.83204900 -0.94927800 -0.44666900
C -7.98892500 1.03574100 -0.51220100
H -8.95858500 1.51099000 -0.32967400
H -7.26389600 1.63317500 0.04246400
C -7.71978600 1.15100500 -2.02217500
H -7.88690700 2.19392800 -2.31160600
H -8.45637300 0.55230100 -2.57322300
C -6.33569700 0.73903400 -2.52137600
H -6.19020100 -0.34240500 -2.43125700
H -6.24292300 0.98731200 -3.58070100
O -5.28155400 0.52171900 0.77228600
O -5.25630200 1.42360800 -1.86743800
H -5.36422800 1.30792100 -0.90479500
N 2.27737100 0.05470200 -2.29430700
N 4.51731700 -0.21345200 -1.54576400
N 3.63930200 1.95257300 -1.90490600
N 1.43376300 2.19417000 -2.59197600
N 5.88054100 1.68306800 -1.17369500
N 3.17635500 -2.04882800 -1.90953000
C 2.48803600 1.37557000 -2.26626200
C 3.31442400 -0.71496300 -1.91016500
C 4.59942900 1.09753200 -1.57156900
C 6.89193000 0.82548100 -0.95741400
C 5.90552100 3.02249700 -1.03304300
C 0.12191097 1.68739654 -2.96882734
C 1.52058176 3.64059614 -2.53023365
H -0.00419219 0.60981419 -2.98586917
H -0.61979919 2.44164477 -3.20130131
H 0.61873432 4.17086374 -2.81277726
H 2.46744320 4.06190074 -2.21102676
H 5.03365500 3.58406400 -1.29840300

H 6.83700800 3.46933300 -0.72806800
H 7.84009300 1.24283100 -0.66249700
H 6.70986100 -0.22472000 -1.06038700
H 3.97576900 -2.52804400 -1.51102300
C 1.91465000 -2.83099600 -1.82285000
C 2.35884900 -4.29519500 -1.65476300
C 1.14279400 -2.45679100 -0.53529200
C 2.39611100 -4.08187400 0.73142200
C 1.23933100 -5.04008700 1.02338100
C 3.42274600 -4.07341100 1.84765300
O 3.10725600 -4.46259800 -0.45585700
O 1.94127900 -2.73820400 0.60961500
C 0.93925500 -2.82594700 -3.02097500
O 0.75543000 -1.66775700 -3.56294200
H 1.25139800 -0.90097800 -3.11912100
H 0.94772800 -1.38943300 -0.48716400
H 0.18077900 -2.97056600 -0.51119500
H 3.01379200 -4.59829300 -2.47309500
H 1.46835900 -4.92491900 -1.67968600
H 4.24543500 -3.41016900 1.57909900
H 2.96506200 -3.71198000 2.76892500
H 3.81394800 -5.07943500 2.00453500
H 0.77110100 -4.75666400 1.96716500
H 0.47009900 -5.02160700 0.25186000
H 1.62017600 -6.05932700 1.10558200
O -3.66860100 -0.75341100 -1.63072900
C -2.37050500 -0.93568500 -1.32616700
C -1.40241400 0.18589500 -1.65757800
H -0.47193900 -0.05139200 -1.14620500
H -1.20942400 0.10515000 -2.73328500
C -1.82278200 1.61579600 -1.29830800
H -0.97841800 2.25915500 -1.56472900
H -2.67266500 1.94589500 -1.90623300
C -2.15384700 1.80583900 0.18643500
H -3.13367900 1.38103600 0.40292400
H -1.42617600 1.26244400 0.79662200
C -2.14457200 3.28434800 0.59814100
H -2.50850700 3.90830700 -0.22636500
H -2.81755000 3.45596200 1.44226800
C -0.76136000 3.76640200 1.00295000
H -0.73022900 4.85662900 1.08845700
H -0.00387300 3.44048000 0.28425800
O -0.49216000 3.18392200 2.29736300
C 0.70453800 3.28817000 2.91659800

C 1.84625300 3.99874700 2.21376200
H 2.37991800 4.56909300 2.97728100
H 1.48487500 4.70187400 1.46401700
C 2.80769800 2.97423400 1.56574100
H 3.45920500 3.49565900 0.85906000
H 2.21850300 2.26370700 0.98088100
C 3.65867600 2.24177900 2.61010600
H 4.38004800 2.95047800 3.03116100
H 3.02354500 1.92920600 3.44079700
C 4.41643100 1.02108000 2.07992300
H 5.03971800 0.61516800 2.88287100
H 5.09795200 1.30266200 1.27170400
C 3.50525500 -0.08415900 1.56499800
O -2.01916700 -1.98599100 -0.84335600
O 0.82781200 2.77206900 4.00246500
H -3.91182300 0.16747600 -1.87167700
O 2.59274600 -0.48614400 2.61238700
O 0.79262400 0.18100200 1.41944300
H 2.91616100 0.23244300 0.70853300
H 4.06669200 -0.97460900 1.28929300

Coordinates of equilibrated DOD:PCL model complexes

2

C -3.44757400 2.81360100 -0.66495100
C -4.79625000 3.43464500 -0.94865500
H -5.23576700 3.78298500 -0.01245900
H -4.59625800 4.31578700 -1.56766400
C -5.76548700 2.50475800 -1.69871900
H -6.59737500 3.11828500 -2.05606100
H -5.26358600 2.10646900 -2.58570000
C -6.32636000 1.35565300 -0.84711400
H -6.68592800 1.76351700 0.10590100
H -7.20424100 0.93735500 -1.35214200
C -5.31885800 0.23496100 -0.57693000
H -5.05992100 -0.26876600 -1.51350500
H -4.39870100 0.64533300 -0.16843400
C -5.83939400 -0.78916500 0.42032100
H -6.72960400 -1.30087800 0.05300200
H -6.06266300 -0.31605400 1.37888500
O -4.87700400 -1.84979100 0.63400400
C -3.79405200 -1.53672100 1.37883600
C -2.83003500 -2.68846100 1.49231700
H -1.83811900 -2.29055800 1.27319800
H -3.06539100 -3.42529400 0.72647700

Coordinates of equilibrated DOD:PCL model complexes

3

C 5.10132100 -2.75214700 -0.56189900
C 3.85420100 -3.04648300 -1.35742000
H 3.18973500 -2.18256800 -1.25380100
H 4.13439300 -3.06079800 -2.41412500
C 3.11319700 -4.33437900 -0.98655900
H 2.28601900 -4.43388500 -1.69895000
H 3.77013600 -5.19361000 -1.15386100
C 2.54795400 -4.41075500 0.43928700
H 1.89395700 -5.28677700 0.49666900
H 3.36501600 -4.57157200 1.14600700
C 1.76670300 -3.16517700 0.86580400
H 2.45467400 -2.33215600 1.03779200
H 1.08343100 -2.84003500 0.07435900
C 0.96557000 -3.36835700 2.13792600
H 0.52901400 -2.44035700 2.50189200
H 1.56525300 -3.82719800 2.92571400
O -0.11164900 -4.31723200 1.87349500
C -1.36459300 -3.85988300 1.81392300
C -2.33675400 -4.93797700 1.38417000
H -2.85159400 -5.25580300 2.29765300
H -1.77475900 -5.79764800 1.01461900

C -2.83400100 -3.34996300 2.88647100
H -2.12341500 -4.18012100 2.84040100
H -3.81505500 -3.80120400 3.06797400
C -2.48073800 -2.42244800 4.06242600
H -3.32969700 -1.76518900 4.27100200
H -2.34571400 -3.04337100 4.95617000
C -1.24659600 -1.54031200 3.83955700
H -1.09645300 -0.89636900 4.71226600
H -1.44343100 -0.87248000 2.99898200
C 0.04987100 -2.29897300 3.56276500
H 0.41280800 -2.79206900 4.46914000
H -0.10382900 -3.08156700 2.80938400
O -3.64115200 -0.44599600 1.88658700
O 1.08886700 -1.42126800 3.13129400
H 0.83085800 -1.11863500 2.24410700
N 2.11320500 0.93970200 -1.99567500
N 0.87932000 2.93345000 -1.56060400
N 3.25845300 2.94392300 -1.51209700
N 4.47753400 0.93333000 -1.83722000
N 2.06085600 4.91263800 -1.27888600
N -0.20692000 0.93415300 -1.89287100
C 3.19752100 1.65534700 -1.78279700
C 0.95583200 1.61324900 -1.82237900
C 2.05311000 3.54346700 -1.44745700
C 0.85365929 5.70596314 -1.19286137
C 3.28547383 5.67488243 -1.12920188
C 5.57924200 1.64918300 -1.55097900
C 4.42320300 -0.37745600 -2.12893600
H 6.52577500 1.13499100 -1.58716300
H 5.47916300 2.70491700 -1.39945400
H 5.35948000 -0.90495500 -2.20643500
H 3.47323900 -0.83651800 -2.30695700
H 4.20814164 5.10799556 -1.05609227
H 3.34474537 6.59014322 -1.70668588
H -0.08989982 5.17200991 -1.18342839
H 0.86053130 6.62940710 -1.76058966
H -1.04127300 1.49341200 -1.72016700
C -0.33173400 -0.51698100 -1.77355700
C -1.82748900 -0.83494000 -1.63046300
C 0.14770100 -1.21518900 -3.05503600
C -1.53128400 -2.94491100 -2.76226300
C -1.55017800 -4.41352300 -2.38215500
C -2.35767600 -2.65196600 -4.01452000
O -2.02938400 -2.24350500 -1.63123200
C -3.38379600 -4.45288500 0.36831500
H -3.80265700 -3.51376600 0.73202200
H -4.20777200 -5.17248000 0.36359700
C -2.87612500 -4.27951700 -1.07237800
H -2.73737500 -5.26269200 -1.53716900
H -3.67853000 -3.79369000 -1.64151700
C -1.57618500 -3.47383900 -1.21838300
H -0.71551600 -4.12779100 -1.04262900
H -1.52388400 -2.68421000 -0.46931400
C -1.40437800 -2.80440100 -2.57875800
H -0.38403500 -2.43210700 -2.67742000
H -1.58929700 -3.51081000 -3.39812200
O -1.67778500 -2.71660400 2.09056800
O -2.23636900 -1.64518900 -2.72755900
H -3.16377800 -1.88046600 -2.85317100
N -1.62451600 0.82231400 1.57123300
N -0.46534100 -0.22851700 -0.22707800
N 0.75454700 0.75885000 1.53636400
N -0.37890400 1.83665700 3.25357200
N 1.87871200 -0.00785200 -0.40333000
N -2.73886900 -0.34922800 -0.12001200
C -0.42461600 1.09713400 2.08880700
C -1.60866100 0.09750600 0.44238600
C 0.63596100 0.15901300 0.35847300
C 1.74251300 -0.22792000 -1.72219200
C 3.02832800 0.10847700 0.28098300
C -1.64501334 2.23907656 3.79492456
C 0.93847100 2.20586400 3.77835200
H -1.73045966 2.82620170 4.70619712
H -2.60462034 2.00796676 3.34580223
H 1.44486600 2.93578900 3.14006400
H 0.79688200 2.62949000 4.77294200
H 2.98105500 0.38483100 1.31512100
H 3.94170000 0.02847400 -0.27854300
H 2.63476700 -0.19676200 -2.32447100
H 0.75199000 -0.30497600 -2.12499900
H -2.59111300 -0.74024100 -1.05293300
C -4.11938600 -0.01313100 0.24414000
C -4.42294500 1.44435400 -0.12970100
C -5.02332700 -0.88327000 -0.63805100
C -4.99660600 0.79241700 -2.37993700
C -6.47928500 1.16993900 -2.35251600
C -4.39136400 0.93413600 -3.76350300
O -4.22820600 1.63713000 -1.52998700

O -0.14650000 -2.61728800 -2.98583100
O 0.09831300 -0.36913900 0.64343800
H 0.71910100 0.37666300 0.71270500
H -0.33256700 -0.75756500 -3.92524200
H 1.22525000 -1.13477800 -3.17231600
H -2.39926400 -0.34219700 -2.42378000
H -2.20073300 -0.47921700 -0.67278600
H -1.95994700 -3.21867100 -4.85774200
H -3.39228300 -2.95093200 -3.83901800
H -2.35071300 -1.59474100 -4.28017500
H -0.94034200 -4.56354800 -1.49007300
H -2.57390600 -4.72556100 -2.17175500
H -1.15234200 -5.01766100 -3.19899500
O 1.61696800 -3.99665100 -0.06061600
C 2.50429600 -3.84256600 -0.87043200
C 3.97841500 -4.05253900 -0.60675800
H 4.17918900 -5.07023200 -0.96278300
H 4.54487400 -3.39065000 -1.26560100
C 4.42306700 -3.91087100 0.85629700
H 3.58670000 -4.16863400 1.51210300
H 5.20879100 -4.64790500 1.04421700
C 4.97793300 -2.52577500 1.22144000
H 5.56675700 -2.61966800 2.14128100
H 5.68168300 -2.21058400 0.43978900
C 3.92625600 -1.43223200 1.44067000
H 3.25797000 -1.35991100 0.58216500
H 3.30927400 -1.67918800 2.30791700
C 4.61358600 -0.08684200 1.66407400
H 4.80443300 0.44207000 0.72957100
H 5.55783000 -0.22054800 2.19234200
O 3.88796100 0.81605000 2.55787000
C 2.78014100 1.39113700 2.11535800
C 2.21645200 2.37933200 3.11212100
H 2.97630700 3.16072100 3.22325500
H 2.15504200 1.88159100 4.08408600
C 0.87069700 2.97424200 2.69002800
H 0.89755600 3.15070500 1.61215600
H 0.74743200 3.95152500 3.16801300
C -0.32663000 2.08804600 3.05328900
H -0.15813600 1.06531400 2.71190400
H -0.39298200 2.02310000 4.14408400
C -1.66716500 2.59789500 2.50722800
H -1.78389800 3.66420400 2.73256700
H -2.48784300 2.06946300 2.99858200
O -4.82282100 -0.59169500 -2.01959700
O -4.29668200 -1.64542900 2.07906100
H -3.35351600 -1.88096300 2.12394500
H -4.79466900 -1.93860800 -0.51019900
H -6.06696600 -0.72334200 -0.34683900
H -3.75309100 2.13456800 0.37308400
H -5.44733000 1.69951100 0.16669600
H -4.46344200 1.97148800 -4.09305400
H -3.34264800 0.63836800 -3.72808500
H -4.92280300 0.29639300 -4.47192100
H -7.03256400 0.52886800 -3.04070500
H -6.92022400 1.06404000 -1.36093900
H -6.59315500 2.20969800 -2.66324500
O -3.32843400 3.93678400 1.77364700
C -2.16436600 4.19890200 1.59228500
C -1.57889400 4.82874800 0.35562900
H -2.30148600 5.52880800 -0.06657400
H -0.67335300 5.37125800 0.63101700
C -1.25275900 3.71431400 -0.66720700
H -0.79794900 2.87125200 -0.14357300
H -2.18604600 3.33749800 -1.09219500
C -0.32315500 4.16818600 -1.80072800
H -0.28249100 3.36506800 -2.54420200
H -0.76211500 5.03446600 -2.30767600
C 1.11267200 4.51397900 -1.37355300
H 1.68351700 4.80478300 -2.26013300
H 1.12468900 5.37345500 -0.69540600
C 1.81649700 3.33924300 -0.70849600
H 1.81603500 2.46623500 -1.36116700
H 1.36506600 3.07740800 0.24631800
O 3.18563100 3.67705900 -0.37281400
C 4.15232200 3.26674000 -1.21944300
C 5.52345900 3.56641500 -0.65882800
H 5.46704500 4.46949200 -0.04886300
H 6.19840500 3.74678700 -1.49824600
C 6.04306900 2.39164800 0.19657500
H 5.28346800 2.13734300 0.94316600
H 6.92319100 2.73326900 0.75189200
C 6.40456400 1.14787500 -0.61843700
H 5.57005200 0.87501200 -1.26785700
H 7.23885300 1.38413900 -1.29039000
C 6.78625200 -0.04303400 0.26505600
H 6.00418700 -0.23274400 1.00799900
H 7.69767900 0.18763200 0.82989500

C -1.80474400 2.38002500 1.01191600
O 2.28233700 -3.50823400 -2.15261500
O 2.30541900 1.17850700 1.01255000
H 1.31853400 -3.39187100 -2.32225800
O -3.07970600 2.96080700 0.60652600
O -2.77503500 2.25157700 -1.50914800
H -1.01512700 2.85708300 0.42832400
H -1.81622900 1.31610000 0.78096600
C 0.42620300 -1.07524000 -0.55140000
H 1.49862600 -1.02547800 -0.73148500
H 0.14692700 -2.11367700 -0.40201400

Coordinates of equilibrated DOD:PCL model complexes

4

C -0.81411700 0.24752600 3.48041000
C -1.70346100 1.40143000 3.82817900
H -1.23896000 1.94172800 4.66129800
H -1.67480500 2.09786000 2.96065700
C -3.14216200 1.00306100 4.16018700
H -3.15654000 0.20489300 4.90991900
H -3.63402700 1.87295700 4.61021900
C -3.92987300 0.56855500 2.92242200
H -3.82866900 1.34258900 2.15826700
H -3.47754300 -0.33765000 2.51583200
C -5.41245500 0.33003200 3.20447300
H -5.53760400 -0.40212600 4.01035900
H -5.87760600 1.26111700 3.55175500
C -6.19912700 -0.14219600 1.99098700
H -6.02882300 0.49271700 1.12155900
H -7.26760300 -0.18704400 2.20535800
O -5.83832300 -1.51011000 1.65287200
C -5.05504300 -1.73904900 0.58770100
C -4.83500100 -3.21872900 0.37700500
H -5.53092800 -3.51478400 -0.41709300
H -5.13501700 -3.75096100 1.28166600
C -3.40134000 -3.57346400 -0.02825200
H -3.13165600 -3.03858800 -0.94119900
H -3.37935900 -4.64439700 -0.26188400
C -2.36835200 -3.27193400 1.06254300
H -2.22082800 -2.19107400 1.14332300
H -2.74947200 -3.60339100 2.03713500
C -1.02872200 -3.95963000 0.79333000
H -0.64191700 -3.66686000 -0.18638200
H -1.18496900 -5.04614700 0.75231700

C 7.03355900 -1.32582900 -0.51540700
O -1.17804400 3.87773900 2.46860100
O 3.93960500 2.69884400 -2.26569600
H 1.56304800 1.31937000 3.85372900
O 5.81927200 -1.77306800 -1.16742200
O 5.43980500 -3.29005800 0.46571400
H 7.74398500 -1.16337300 -1.32821300
H 7.39512900 -2.12199800 0.13419500
C -4.43809500 -0.28331000 1.72488600
H -5.48703100 -0.02462100 1.89308900
H -3.82217600 0.36134500 2.35219900

Coordinates of equilibrated DOD:PCL model complexes

5

C -1.56127018 -4.64832388 2.12731671
C -0.59581736 -3.51983418 2.36488272
H -0.94237109 -2.64589675 1.78998221
H -0.66577345 -3.24220581 3.41810173
C 0.84808249 -3.85806681 1.99913016
H 1.48059259 -3.02878847 2.32517749
H 1.15456474 -4.75238593 2.54622194
C 1.07203302 -4.10327767 0.50709808
H 2.12690101 -4.34307906 0.35338179
H 0.48492773 -4.96547179 0.19363587
C 0.70701731 -2.89330450 -0.35150184
H -0.37419352 -2.81059242 -0.44673606
H 1.08115842 -1.97891223 0.11499793
C 1.28324837 -3.00067327 -1.75986749
H 1.00912583 -2.12137891 -2.35163245
H 0.93701270 -3.91007596 -2.25507557
O 2.70857894 -3.11215147 -1.71447526
C 3.43736027 -2.00536861 -1.77847328
C 4.90904193 -2.33727068 -1.62230114
H 5.30192288 -2.48124332 -2.63230201
H 5.00493606 -3.28143940 -1.08690917
C 5.67936128 -1.21018881 -0.93630074
H 5.32446398 -0.27043076 -1.36449253
H 6.74082763 -1.30216943 -1.17425413
C 5.52760346 -1.18888738 0.58806583
H 6.05797463 -2.04542954 1.01231778
H 6.01741131 -0.28482859 0.96111916
C 4.07540862 -1.22435402 1.07089614
H 3.67695619 -2.23189594 0.94623171
H 3.46031627 -0.54719171 0.47720505

C 0.03699700 -3.68444700 1.84546000
H -0.33633200 -3.92071700 2.84868300
H 0.91475800 -4.31043000 1.66051600
O -4.61301200 -0.86645200 -0.12873000
O 0.49247400 -2.32556100 1.81720000
H -0.05506300 -1.77749400 2.42767700
N -0.06635100 0.28108300 -2.17739200
N -1.56198000 -1.57824100 -2.27065300
N 0.74521200 -1.86500500 -2.80965800
N 2.17002300 0.01158800 -2.68221800
N -0.72938400 -3.65843800 -2.85685300
N -2.28213500 0.46750200 -1.48904000
C 0.84224400 -0.57119900 -2.55692400
C -1.29966100 -0.27449100 -1.97396100
C -0.52957900 -2.30737800 -2.65304500
C -1.87087500 -4.16981800 -3.58414200
C -0.47962400 -4.31013200 -4.13044800
C 2.26119500 1.47978200 -2.75798700
C 3.21121100 -0.73255700 -2.56521700
H 1.69173900 1.82029900 -3.62105300
H 3.31079400 1.74739800 -2.84441300
H 4.18918500 -0.26705500 -2.57133000
H 3.05591900 -1.79926300 -2.50399100
H -0.19925700 -3.67387300 -4.96380700
H 0.03513900 -5.26258900 -4.07682500
H -2.52715800 -3.43746900 -4.04196800
H -2.36467200 -5.02333800 -3.13416200
H -3.13606900 -0.05079200 -1.29544300
C -2.17100200 1.80119700 -0.86506500
C -1.58004600 2.86405600 -1.79037300
C -3.58137000 2.25819100 -0.50171500
C -3.75720400 3.60258400 -2.48148000
C -3.72941300 4.96961200 -1.78529300
C -4.58498400 3.63443700 -3.75430700
O -2.44951000 3.19792200 -2.87634600
O -4.35525800 2.61233700 -1.65259800
O -1.26623200 2.81235500 1.18150000
H 1.81738800 1.90292300 -1.85990400
H -4.13411100 1.45526300 -0.01971200
H -3.47678700 3.09138200 0.19618700
H -0.65823000 2.52260100 -2.24845700
H -1.36485900 3.73022500 -1.15843900
H -4.57492900 2.64331200 -4.20907200
H -5.61463200 3.91247700 -3.52339000

C 3.93627189 -0.82778253 2.54480127
H 2.99746457 -1.21538465 2.94888388
H 4.76475673 -1.25747404 3.12606186
O 2.99608066 -0.89784456 -1.96948530
O 3.87018688 0.56411810 2.75196004
H 4.62226974 1.00257815 2.31933182
N 1.46984338 1.88556083 -1.29694486
N 0.39469076 0.54646504 0.35036924
N -0.43423177 0.66014022 -1.85701129
N 0.64793061 1.99711492 -3.52278627
N -1.63588088 -0.52808256 -0.24345488
N 2.34185157 1.84122643 0.73903845
C 0.54649031 1.44654202 -2.21711349
C 1.33397600 1.34260691 0.00140765
C -0.51637391 0.23829649 -0.61485701
C -1.81673471 -0.76323045 1.05558359
C -2.44502037 -0.93915380 -1.21665457
C -0.20315077 3.16430213 -3.65974919
C 0.42640877 0.99263367 -4.55858452
H -0.20213160 3.49112140 -4.70169540
H 0.17604966 3.95828172 -3.00299781
H -0.59394086 0.60279788 -4.54483207
H 0.63253474 1.44482472 -5.52771576
H -2.12597188 -0.85229616 -2.23270639
H -3.31161723 -1.51258374 -0.95334391
H -2.71614959 -1.26675522 1.35575208
H -1.22448341 -0.22279802 1.76230437
H 2.60251082 1.50113967 1.66148056
C 3.15088983 2.78959735 -0.00120838
C 3.10056780 4.19276078 0.65435754
C 4.64415386 2.40160012 -0.06480840
C 5.15554348 3.79420755 1.80769475
C 6.04327576 4.83145149 1.10464487
C 5.60251863 3.60107219 3.25719859
O 3.80784381 4.20338829 1.87249497
O 5.27422470 2.51447223 1.19258480
C 2.48907453 2.71642548 -1.34327897
O 2.96744497 3.30767293 -2.46996832
H 2.43880007 2.96460143 -3.21269568
H 4.74547678 1.35803138 -0.35855778
H 5.13165918 3.03083959 -0.81933021
H 2.07224822 4.45258135 0.91174757
H 3.49380126 4.93157134 -0.05608170
H 5.56415860 4.55236562 3.77655130

H -4.16411800 4.35655800 -4.45604000
H -3.30193700 5.71298800 -2.46122900
H -4.74802800 5.26677100 -1.52783800
H -3.13615200 4.95812100 -0.87103700
O 3.02943100 -2.28855200 1.70898000
C 3.60220300 -1.70820900 0.68337700
C 5.08202000 -1.99448700 0.56794200
H 5.27476800 -3.04301300 0.80664000
H 5.36309600 -1.81699100 -0.47062700
C 5.90265900 -1.08801200 1.50703800
H 5.51332300 -0.07077500 1.46963600
H 5.76053400 -1.43318700 2.53419000
C 7.40427500 -1.08500800 1.17570800
H 7.93824900 -0.54725900 1.96705600
H 7.76822400 -2.11778200 1.21235000
C 7.78796100 -0.48455000 -0.18851500
H 8.79537100 -0.82346200 -0.45493100
H 7.13007200 -0.85519700 -0.97710600
C 7.83557000 1.03790800 -0.25312600
H 8.05956300 1.37502400 -1.26613400
H 8.58157400 1.43479300 0.43581600
O 6.60496000 1.66953300 0.18621800
C 5.55898200 1.66876500 -0.64706400
C 4.29825100 2.20814300 -0.01870800
H 3.61474600 1.35321400 -0.01810800
H 3.87447700 2.93063900 -0.72162100
C 4.39379500 2.81604200 1.38228500
H 4.91652600 3.77541400 1.32877400
H 5.00132700 2.17189700 2.02311800
C 3.01101200 2.99960700 2.02302400
H 3.12783100 3.57778900 2.94617400
H 2.36288400 3.59394600 1.36869200
C 2.33277100 1.66415400 2.33956000
H 2.99414400 1.05245500 2.96524000
H 2.15062000 1.09293400 1.42601000
C 0.99539200 1.83860400 3.03124400
O 3.01930200 -0.96883100 -0.10517800
O 5.61076300 1.23095200 -1.78261300
H 2.01281100 -2.15207400 1.74379000
O 0.47100500 0.48980500 3.19452900
O -1.17316300 -0.92306800 3.43650900
H 1.11105100 2.28881700 4.02309900
H 0.29677300 2.42397400 2.41851100
C -1.30382900 1.68517000 0.44799800
H 4.92757802 2.90418560 3.74510984
H 6.61351904 3.20703855 3.27861777
H 7.07617097 4.49685349 1.13643945
H 5.75447737 4.97395996 0.06707489
H 5.95305895 5.78099496 1.62368422
O -1.15774141 3.54396990 -1.20185918
C -1.88532123 3.03675995 -2.01602303
C -3.26387194 2.50101656 -1.75485472
H -3.91231161 2.81569436 -2.57442831
H -3.18551276 1.40755213 -1.78786027
C -3.80401894 2.96738885 -0.40410792
H -3.28873306 2.43249294 0.39339842
H -3.56743184 4.02639618 -0.28062373
C -5.31708418 2.79232543 -0.27926628
H -5.63120119 3.15530890 0.70130723
H -5.80571981 3.41454395 -1.03267822
C -5.80945434 1.35742955 -0.45527093
H -6.90174964 1.35419386 -0.42446082
H -5.49907183 0.96442545 -1.42451049
C -5.30256771 0.42194447 0.63834643
H -5.64909839 0.76056176 1.62013913
H -4.21559408 0.35045493 0.62784997
O -5.77445167 -0.90745730 0.41004010
C -6.96712319 -1.22453154 0.91329643
C -7.41691179 -2.61155290 0.50636750
H -7.98615581 -2.48595494 -0.41951067
H -8.10960873 -2.96758788 1.27109726
C -6.29211266 -3.61859208 0.27990370
H -5.61957190 -3.24723599 -0.49395309
H -6.73730971 -4.54942627 -0.08017216
C -5.50024501 -3.90655675 1.55398327
H -4.88881984 -3.04146729 1.81367638
H -6.19321090 -4.08494235 2.38001601
C -4.60336795 -5.13089198 1.38610056
H -3.94332940 -4.99635565 0.52685184
H -5.21811853 -6.01427052 1.20278832
C -3.74346117 -5.39282302 2.62018545
O -1.55726628 2.90071304 -3.31848196
O -7.63211166 -0.48312071 1.59075773
H 1.12452054 0.17656113 -4.38411110
O -2.71862015 -4.41339875 2.76804001
O -1.35922335 -5.63943096 1.47273937
H -4.34082372 -5.33238963 3.53256099
H -3.27384012 -6.37965083 2.54536094

H -0.30427600 1.33108200 0.09530700

H -1.73290600 0.79613400 0.97651600

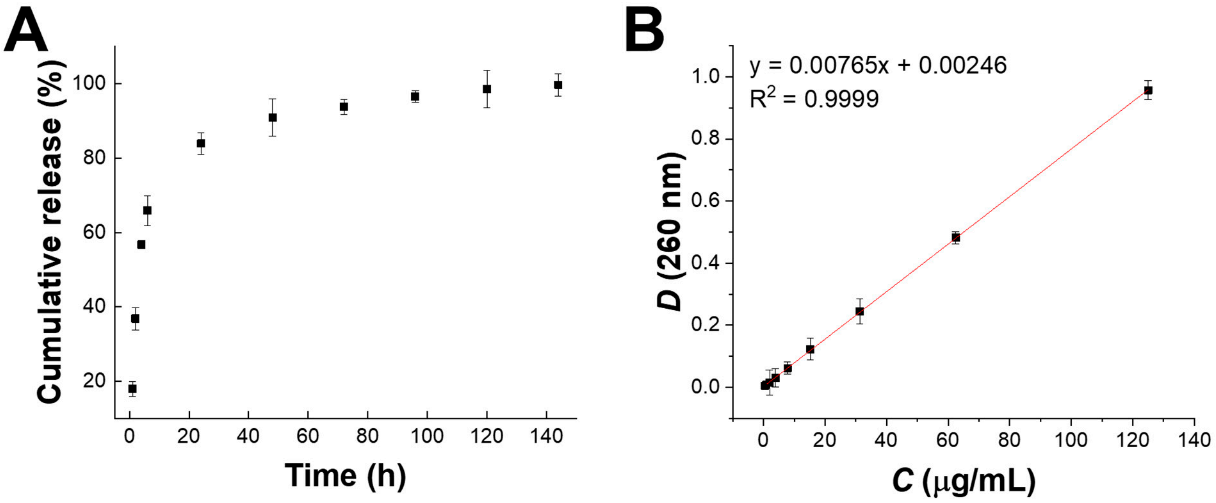

Figure S3. (A) Profile of free DOD release in 0.01 M PBS (pH 7.4) (**B**) The calibration plots for DOD PBS.

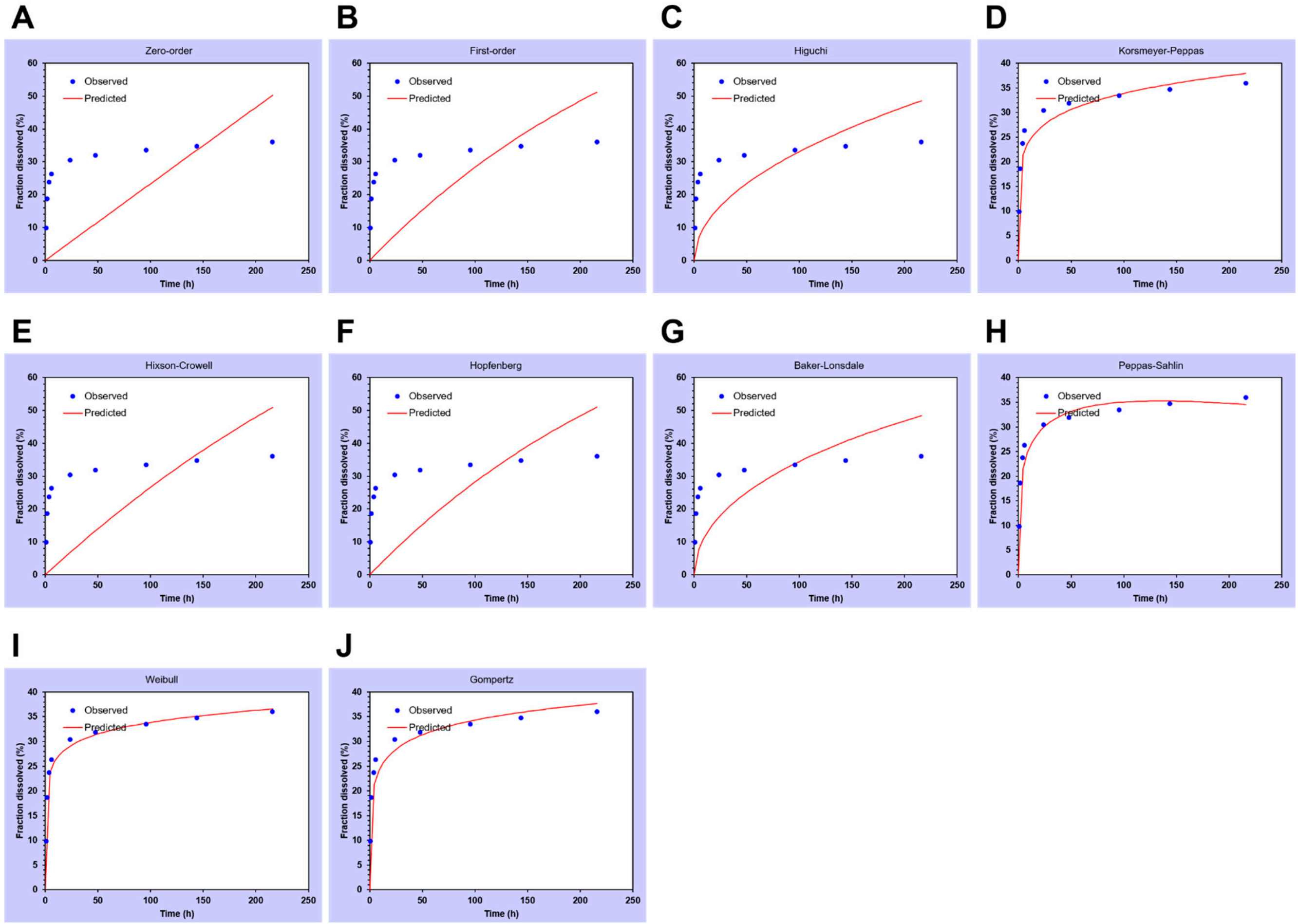

Figure S4. (A) Zero-order; **(B)** First-order; **(C)** Higuchi; **(D)** Korsmeyer-Peppas; **(E)** Hixson-Crowell; **(F)** Hopfenberg; **(G)** Baker-Lonsdale; **(H)** Peppas-Sahlin; **(I)** Weibull; **(J)** Gompertz.

Table S1. Dynamics in body weight of BALB/c mice after tumor grafting and treatment.

Group		Animals body weight after tumor grafting (g); *p* value regarding control					
		2 days	8 days	12 days	14 days	19 days	23 days
I.1	Control	21.9 ± 0.3	27.0 ± 0.5; 0.0002	31.0 ± 0.6; 0.0002	34.6 ± 0.6; 0.0007	41.0 ± 0.9; 0.0007	45.8 ± 2.2; 0.0008
I.2	MTD	22.8 ± 0.3	22.6 ± 0.4; > 0.05	24.3 ± 0.6; > 0.05	28.4 ± 1.0; < 0.0001	31.9 ± 1.5; < 0.0001	35.1 ± 2.2; < 0.0001
I.3	MTD/NPs	24.5 ± 0.2	26.1 ± 0.5; 0.0225	29.9 ± 1.0; 0.0005	32.8 ± 1.1; < 0.0001	39.6 ± 1.4; < 0.0001	43.2 ± 1.9; < 0.0001
I.4	2MTD/NPs	24.7 ± 0.2	25.2 ± 0.5; > 0.05	28.2 ± 0.8; 0.0019	31.5 ± 1.2; 0.0012	36.6 ± 1.9; 0.0011	40.2 ± 3.2; 0.0009
I.5	4MTD/NPs	24.8 ± 0.3	20.3 ± 0.9; 0.0286	24.5 ± 1.5; > 0.05	28.5 ± 1.5; > 0.05	31.8 ± 0.3; 0.0286	32.0 ± 2.1; 0.0286

Table S2. Dynamics in body weight of DBA mice with L1210 lymphoid leukemia after tumor grafting and treatment.

Group		Body weight (g); wight dynamics (%) *				
		2 days	6 days	9 days	12 days	16 days
II.1	Control	26.5 ± 0.8; 100	29.6 ± 1.0; 112	–	–	–
II.2	MTD	25.3 ± 0.8; 100	24.9 ± 0.9; 98	26.8 ± 1.0; 106	26.3 ± 0.8; 104	–
II.3	3MTD/NPs	20.6 ± 0.6; 100	17.5 ± 0.6; 85	19.6 ± 0.6; 95	21.0 ± 0.6; 102	22.2 ± 0.6; 108

* $p < 0.05$ compared to the initial body weight of the animals.

Table S3. Dynamics in tumor growth in C57Bl/6 mice grafted with Lewis lung carcinoma.

Group		Tumor volume (mm^3); Tumor growth inhibition index (%); *p* value regarding control					
		**20 days**	**24 days**	**28 days**	**31 days**	**35 days**	**38 days**
I.1	Control	915 ± 482	1786 ± 483	2907 ± 488	4175 ± 1333	5501 ± 1534	6436 ± 1795
I.2	MTD	975 ± 336;<br>–;<br>0.8975	1498 ± 440;<br>16;<br>0.5371	3154 ± 792;<br>–;<br>0.8977	3315 ± 901;<br>21;<br>0.9138	3876 ± 940;<br>29.5;<br>0.7879	4866 ± 1577;<br>24;<br>0.8452
I.3	3MTD/NPs	70 ± 52;<br>92;<br>0.0150	527 ± 376;<br>70.5;<br>0.0202	968 ± 513;<br>67;<br>0.0086	1200 ± 577;<br>71;<br>0.0220	2040 ± 1011;<br>63;<br>0.0252	3186 ± 1118;<br>50.5;<br>0.2091
I.4	4MTD/NPs	495 ± 395;<br>46;<br>0.2316	1087 ± 558;<br>39;<br>0.2879	1369 ± 757;<br>53;<br>0.1688	1845 ± 895;<br>56;<br>0.3052	2261 ± 1391;<br>59;<br>0.1349	3037 ± 1277;<br>53;<br>0.1786
